# Design and development of novel, short, stable dynorphin-based opioid agonists for safer analgesic therapy

**DOI:** 10.3389/fphar.2023.1150313

**Published:** 2023-03-03

**Authors:** Rink-Jan Lohman, Karnaker Reddy Tupally, Ajit Kandale, Peter J. Cabot, Harendra S. Parekh

**Affiliations:** School of Pharmacy, Pharmacy Australia Centre of Excellence, The University of Queensland, Woolloongabba, QLD, Australia

**Keywords:** dynorphin, kappa opioid receptors, biased agonists, non-addictive, pain treatment

## Abstract

Kappa opioid receptors have exceptional potential as an analgesic target, seemingly devoid of many problematic Mu receptor side-effects. Kappa-selective, small molecule pharmaceutical agents have been developed, but centrally mediated side-effects limit clinical translation. We modify endogenous dynorphin peptides to improve drug-likeness and develop safer KOP receptor agonists for clinical use. Using rational, iterative design, we developed a series of potent, selective, and metabolically stable peptides from dynorphin 1–7. Peptides were assessed for *in vitro* cAMP-modulation against three opioid receptors, metabolic stability, KOP receptor selectivity, desensitisation and pERK-signalling capability. Lead peptides were evaluated for *in vivo* efficacy in a rat model of inflammatory nociception. A library of peptides was synthesised and assessed for pharmacological and metabolic stability. Promising peptide candidates showed low nanomolar KOP receptor selectivity in cAMP assay, and improved plasma and trypsin stability. Selected peptides showed bias towards cAMP signalling over pERK activity, also demonstrating reduced desensitisation. *In vivo*, two peptides showed significant opioid-like antinociception comparable to morphine and U50844H. These highly potent and metabolically stable peptides are promising opioid analgesic leads for clinical translation. Since they are somewhat biased peptide Kappa agonists they may lack many significant side-effects, such as tolerance, addiction, sedation, and euphoria/dysphoria, common to opioid analgesics.

## 1 Introduction

Despite demonstrating potent analgesia, mainstay opioid-based analgesic therapy produces an array of adverse effects ranging from mild nausea, sedation and itch to severe constipation, respiratory depression, and death. More importantly, the development of psychological addiction, dependence, and tolerance to opioid drugs limit their long-term clinical use (Benyamin et al., 2008). Opioid-related deaths, whether accidental, intentional, through illicit or prescription medications, have become a global pandemic pre-existing COVID-19 and is aptly termed the “Opioid Crisis” (Vadivelu et al., 2018; Coussens et al., 2019; Taha et al., 2019). In 2020, striking figures of over 90,000 deaths in the United States alone were attributed to opioid overdose; approaching 30% higher than in previous years (Lancet, 2021).

Most clinically used analgesic opioids are alkaloids that target the Mu-opioid receptor (MOP receptor), and the most prominent dose-limiting side-effects stem from MOP receptor activation in the central nervous system (CNS) (Hua and Cabot, 2010; Al-Hasani and Bruchas, 2011; Iwaszkiewicz et al., 2013). Like MOP receptor, Kappa opioid receptor (KOP receptor) signalling is well accepted to be involved in nociception (Millan, 1990; Riviere, 2004; Camilleri, 2008; Hughes et al., 2013; Snyder et al., 2018). The KOP receptor is highly expressed throughout the central and peripheral nervous systems (PNS). In the PNS, KOP receptor is present on peptidergic neurons, myelinated A-δ and A-β fibres and unmyelinated c-fibres terminating in skin, hair follicles and viscera (Snyder et al., 2018). Functionally, these reduce nociception and neurogenic (afferent-fibre) plasma extravasation (Snyder et al., 2018). KOP receptor activation on primary afferents in the dorsal root ganglion seems to lack the most problematic peripherally mediated MOP receptor side-effects, such as gastrointestinal stasis (Riviere, 2004), nausea and itch (Riviere, 2004; Naser and Kuner, 2018), even suppressing MOP receptor-mediated tolerance (Vanderah et al., 2000; Wang et al., 2009). Hence, it is not surprising that KOP receptor is a validated target for itch and visceral and inflammatory pain.

Various established KOP receptor selective agonists (e.g., U50488H, U69593, CR845, and spiradoline (Ur et al., 1997; Webster et al., 2015; Fishbane et al., 2020) have shown remarkable activity as antinociceptives and antipruritics. However, the majority of these compounds show both PNS and distinctly CNS-mediated side-effect profiles (e.g., nausea/vomiting, diuresis, dizziness, sedation, anxiety, dysphoria, hallucinations, and psychosis (Ur et al., 1997; Hasebe et al., 2004; Webster et al., 2015; Fishbane et al., 2020) precluding their clinical use. A major focus for PNS-acting, KOP receptor-targeting analgesic drug discovery is to avoid crossing the blood-brain barrier (Machelska and Celik, 2018; Naser and Kuner, 2018; Snyder et al., 2018) to mitigate CNS mediated side-effects. Another recently highlighted focal point for drug development is identifying bias agonists of KOP receptor (Allouche et al., 2014; Brust et al., 2016; Dunn et al., 2019) favouring Gi-protein (adenylate cyclase reduction of cAMP production) bias over other G-protein pathways, namely, MAPKs, pERK, and β-arrestin. Indeed, biased agonists have been shown to reduce sedation, dysphoria (Brust et al., 2016), desensitisation and tolerance (Allouche et al., 2014). An ideal KOP receptor agonist may require the properties of limited brain permeability [difelikefalin, CR665 (Aldrich and McLaughlin, 2022)] and biased signaling towards only cAMP, as related to nociception, thus bypassing the most problematic side effects.

Endogenous opioid peptides are produced within the CNS, PNS, and the immune system (Lord et al., 1977; Sibinga and Goldstein, 1988; Cabot et al., 1997). Enkephalins target MOP receptor whilst the dynorphins bind to KOP receptor (Wittert et al., 1996). Immune cells (especially leukocytes like neutrophils) provide local delivery of various analgesic endogenous opioid peptides, including β-endorphin, dynorphin (Dyn1-17) and leu-enkephalin (Lord et al., 1977; Sibinga and Goldstein, 1988; Chou et al., 1996; Wittert et al., 1996). Thus, the immune response provides site-directed analgesia in times of inflammation or tissue damage.

If considered on potency and specificity alone, endogenous opioid peptides appear superior analgesics to alkaloids (Chou et al., 1996). However, being peptides, poor intrinsic metabolic stability and bioavailability restrict their clinical viability. Once released, localised bio-metabolism of parent endogenous KOP receptor ligand, Dyn1-17 rapidly (within minutes) generates many fragments (Morgan et al., 2012), and the highly promising lead fragment, Dyn1-7. This heptameric peptide retains the conserved message domain and an apparent minimal requirement of the address domain, retaining the KOP receptor selectivity and potency of Dyn1-7, comparable to U50488H (Morgan et al., 2012; Morgan et al., 2017).

The advantage of peptides in pharmacology is their exquisite selectivity and potency for their target receptor. When considering the physicochemical properties (i.e., molecular weight, lipophilicity, polar surface, volume and flexibility) of such relatively large peptides, it is expected they have low blood-brain barrier (BBB)-permeability (Pajouhesh and Lenz, 2005; Banks, 2015). Noting Dyn1-7 possess ≥6 residues, an *N*-terminus free -NH_2_, and at least 2-Arg residues, these collective attributes intrinsically reduce potential to cross the BBB (Banks, 2015). Therefore, our attention focused on improving instability and bioavailability, which constitute significant bottlenecks in the development of clinically useful agents (Hill et al., 2014; Wang et al., 2014; Bockus et al., 2015; Lipinski, 2016). Recent advances in peptide chemistry have recognised physicochemical alterations (e.g., *N*-methylation, cyclisation, residue substitution, side-chain modification) to parental/endogenous backbone structures that allow vast improvements in metabolic stability, permeability and oral bioavailability (Koch et al., 2001; Kilkenny et al., 2010; Rajagopal et al., 2011; Hill et al., 2014; Wang et al., 2014; Lipinski, 2016; Li et al., 2020) often maintaining potency and selectivity (Li et al., 2020). Here, we hypothesised that such alterations to endogenous Dyn1-7 would improve the suitability as opioid analgesics, potentially making them useful for clinical application.

We used iterative rational modification protocols drawn from key sequence and structural attributes of Dyn1-7 and employing a combination of *N*-methylation, and natural and unnatural amino acid substitution strategies. The aim was to create a library of novel peptides with exquisite KOP receptor selectivity and potency whilst improving their metabolic stability, ideally showing a signaling bias towards cAMP modulation (Morgan et al., 2012; Morgan et al., 2017; Li et al., 2020). Peptides were screened for cAMP modulation in respective opioid receptor overexpressing HEK293 cell lines, metabolic stability in rat plasma and trypsin, KOP receptor binding, pERK recruitment and desensitisation. Selected peptides displayed cAMP bias over pERK. Also, they showed little desensitisation potential *in vitro*, indicating these peptides may have a reduced tendency to develop tolerance *in vivo*. In a rat model of inflammatory mechanical nociception, the selected peptides were found to be as effective as morphine at reversing inflammatory nociception in Freund’s complete adjuvant model of inflammatory nociception, following local administration.

## 2 Materials and methods

### 2.1 Chemicals

All solvents required for synthesis, including ethanol, methanol, acetone, acetonitrile (ACN), tetrahydrofuran (THF), petroleum spirit, chloroform, dichloromethane (DCM), *N*-methyl-2-pyrrolidone (NMP) and dimethylformamide (DMF), were purchased from Merck and ChemSupply Pty (Aust.). Special reagents for the solid phase peptide synthesis (SPPS), i.e., piperidine, diisopropyl carbodiimide (DIC), diisopropylethylamine (DIPEA), acetic anhydride, Oxyma Pure (ethyl cyanohydroxyiminoacetate), trifluoroacetic acid (TFA) and triisopropyl silane (TIPS), were purchased from various suppliers - Chem-Impex Inc., Novabiochem^®^, Sigma-Aldrich Pty Ltd. and Thermo Fisher Scientific. Solid bed support (Rink amide AM and Wang resin) having 0.3–0.6 mmol/g loading, and Fmoc-protected amino acids were purchased from Chem-Impex Inc. and P3Biosystems.

### 2.2 Solid-phase peptide synthesis and characterisation

The series of linear peptides were synthesised using a Biotage^®^ Initiator^+^ Alstra™ instrument. Standard Fmoc solid-phase synthesis was used to prepare all peptides. Synthesis was carried out on Rink amide AM resin (0.60 meq/g). Oxyma Pure (0.5 M) and DIC (0.5 M) were used to sequentially couple each amino acid. All coupling reactions were performed under microwave conditions except for Arg residues, which were performed at room temperature. Fmoc deprotection was carried out using 20% v/v piperidine in DMF. Capping was performed after each amino acid coupling using acetic anhydride (5 M in DMF, 8 eq.) and diisopropylethylamine (DIPEA, 2 M in NMP, 8 eq.). After synthesis, the dry resin was collected, and off-resin cleavage was performed using a TFA cleavage cocktail (TFA: TIPS: H_2_O: DCM, 90:2.5:2.5:5).

Crude peptides were collected and further purified by preparative HPLC. All peptides were purified using an Agilent 1200 Chem Station equipped with a binary pump and auto-fraction collector. A Jupiter C_18,_ 10 μm, Proteo 90 Å LC column 250 mm × 21.2 mm was used with a 10 mL/min flow rate. The mobile phase employed was Solvent A: Milli-Q water Solvent B: ACN, both containing 0.1% v/v TFA with a gradient flow 0%–100% B over 60 min. Peptide purity was determined using a Shimadzu LC-2040C Nexera-i HPLC system. The mobile phase employed was Solvent A: Milli-Q (MQ) water, Solvent B: acetonitrile (ACN), containing 0.1% v/v TFA with a gradient flow of 0%–100% B over 35 min. Peptide mass was confirmed by Agilent 1290 ultra-high performance liquid chromatography system coupled with a quadrupole time of flight (Q-TOF, Agilent 6,520) LCMS system.

### 2.3 *In vitro* assays and *in vivo* assays

#### 2.3.1 Cell assays/cAMP activity

Human embryonic kidney (HEK293, RRID:CVCL_0045) cells stably transfected separately with KOP receptor, MOP receptor, or delta-opioid receptor (DOP receptor) were maintained using standard culture techniques (DMEM, 10% foetal bovine serum). At 90% confluence, cells were harvested in HBSS buffer containing 0.25% EDTA, 0.1% bovine serum albumin (BSA), 5 mM HEPES buffer and 0.5 mM 3-isobutyl-1-methylxanthine (IBMX), as per manufacturer’s instruction (ALPHA Screen cAMP kit, Perkin Elmer). cAMP assays were performed in a ProxiPlate-96 (white opaque 96-shallow well microplate). Respective cells (80 K in 20 μL containing acceptor beads) were added to 20 μL forskolin (50 µM) containing the test compound (final concentrations 10 μM-1 pM) and incubated at 37°C for 1 h. For antagonist experiments, naloxone (100 μM) was added directly to the cell suspension and incubated for 30 min at 37°C before plating onto 96 well plates containing forskolin and test compound. Reactions were stopped using the manufacturer’s lysis buffer and then left at room temperature in a dark humidity box on a rotary shaker table overnight. Plates were read for ALPHA signal using a PerkinElmer Ensight Fluorometric Plate Reader running Kaleido, v1.2 software and analysed using a combination of Microsoft Excel (RRID:SCR_016137) and GraphPad Prism (RRID:SCR_002798)) software packages. All data were normalised (in percentage maximal response) to forskolin (maximal cAMP production) and buffer control (minimum cAMP production). The final activity of each compound was then normalised to the reference compound’s activity in each cell type, i.e., U50488H for KOP receptor, morphine or fentanyl for MOP receptor and SNC80 for DOP receptor. *EC*_*50*_ was determined in GraphPad Prism (v8.3).

#### 2.3.2 Metabolic stability in trypsin and plasma

Whole blood was collected in-house from adult mixed-gender Wistar rats (RRID: RGD_150520162), and plasma was prepared in 0.2% EDTA as per standard practice. Peptides (final 100 μM) were incubated with pooled rat plasma at 37°C. Samples were collected at different time intervals 0, 5, 10, 15, 30, 60, and 120 min. At each time point, collected samples were immediately added to three volumes of cold ACN. This mixture was vortexed for 30 s and then centrifuged at room temperature (13 K rpm, 5 min). The supernatant was taken and directly placed in glass HPLC vials for LCMS analysis. The protocol for the trypsin stability assay was as for the plasma stability assay except for the substrate (bovine pancreatic trypsin 2.5 μg/mL in NH_4_HCO_3_ buffer, approx. pH 8–8.5, 37°C).

Relative concentrations of each peptide from stability assays in plasma or trypsin were analysed using an Agilent binary LC system consisting of an Agilent 1290 Infinity LC pump, Agilent 1,290 auto-sampler coupled with Triple Quad Mass-Spectrometer (MS, model 6460, Agilent Technologies). A bidentate C_18_ HPLC column (Cogent, 100 Å, 4 μM) with a binary solvent gradient composed of Solvent: A 0.1%v/v formic acid in MQ water and Solvent B: 0.1% v/v formic acid in ACN was used for the separation. The MS parameters were optimised for all compounds to obtain the highest signal in positive Total Ion Current (TIC) mode. Data were analysed using a combination of Microsoft Excel and GraphPad Prism software packages. Data was normalised to time point zero (immediately after compound addition) and expressed in percentages from t = 0. Half-lives were calculated in GraphPad Prism software (v8.3).

#### 2.3.3 Receptor binding assay

Binding efficiency was determined using a Homogenous Time-Resolved Fluorescence (HTRF) assay specific for KOP-binding studies (Tag-lite^®^ Opioid KOP Receptor Ligand Binding Assay, CisBio). Experimental protocols, including use of Naltrindole as a reference ligand, were as according to the manufacturer’s instruction. Plates were read on a Tecan Spark Fluorimeter, and data were analysed using a combination of Microsoft Excel and GraphPad Prism software packages. The *K*_*d*_, *IC*_*50*_ and *K*_*i*_ were determined in GraphPad Prism.

#### 2.3.4 Desensitisation assay

Methods are similar to previously described (Koch et al., 2001). Two 25 cm^2^ flasks of HEK293-KOP receptor cells per compound tested were grown to confluence (DMEM/10% FBS). Media was replaced with fresh warmed sterile media (DMEM/10%FBS) in both flasks. One flask received pretreatment of the test peptide or reference/control compound (1 µM) the other acted as control [untreated, no test/refence compound, equivolume vehicle (PBS) only]. Flasks were returned to the incubator for 6 h, following which cells were harvested and tested for cAMP modulation using the same kit and protocol as described above. Cells were treated with the same peptide/compound (to which they were pre-treated) in a concentration range as per original concentration -response assays described above. Data were compared for EC_50_ with and without pre-treatment.

#### 2.3.5 pERK activation for bias signalling determination

Protocols were used as recommended by the pERK kit manufacturer’s instruction (PerkinElmer AlphaLISA SureFire Ultra pERK1/2 assay kit) with minor modifications. HEK293-KOP receptor cells were grown to confluence and prepared at 200 k cells/mL using 0.25%w/v EDTA. Cells were seeded in DMEM/10% FBS, 40 k/well in sterile, black, clear-bottom 96 well plates (one plate per test peptide). The outermost wells were omitted to avoid edge effects. Cells were returned to the incubator with the lid on the plate and left for 48 h. Old media was replaced with 40 μL clear (indicator dye and FBS free) prewarmed HBSS and incubated for 20 min at 37°C. Serially diluted test peptide (6 × 10-fold dilutions; final concentration in well 1–10 μM in HBSS) was added (10 μL) to each respective well at various time points (30, 20, 10, 5, and 0 min). The assay was stopped using the provided lysis buffer, following which plates were placed on a shaker for 10 min at room temperature. The plate was centrifuged (3,700 rpm, 10 min), and supernatant collected. The lysate from each assay well (5 μL) was added to a well of a ProxiPlate-384 Plus, white 384-shallow well microplate, to which 2.5 μL of commercial ‘acceptor bead’ reaction media was added, and the plate left in the dark at room temp for 1 h. Supplier “donor bead” reaction media (2.5 μL) was added to each well, and the plate was left to incubate in a humidity box at room temperature. Plates were read for ALPHA signal using a PerkinElmer Ensight Fluorimetric Plate Reader running Kaleido, v1.2 software, pERK_1/2_ production was recorded in a time and concentration-dependent manner. Total ERK was not measured.

A bias factor was calculated from the cAMP and pERK data, as previously reported (Rajagopal et al., 2011), using the following formulae;

$$\varepsilon_{ref.lig}=\frac{E_{\max}}{EC_{50}}$$

$$\sigma_{response}=\log\left(\frac{\varepsilon_{lig}}{\varepsilon_{ref}}\right)$$

$$\beta_{lig}=\frac{\left(\sigma_{response1-}\sigma_{response2}\right)}{\surd 2}$$

Where **ε** is the efficiency coefficient for reference (*ref*) or test ligand (*lig*), **σ** is the relative response/pathway-specific signal factor, and **β** is the pathway signal bias factor. Response **one** was cAMP; response **two** was pERK. E_max_ (maximal effect recorded per compound) and EC_50_ were derived from cAMP and pERK activity concentration-response curves. In cases where there was no discernible pERK activity, EC_50_ was substituted for the arbitrary value 100 µm in order to calculate a bias factor.

#### 2.3.6 Animals

Ethical approval for all *in vivo* experimental protocols was obtained from the University of Queensland Health Sciences Ethics Committee. All procedures adhered to The Australian Code of Practice for the Use of Animals for Scientific Purposes (2013) and were reported following the ARRIVE guidelines (RRID: SCR_018719) (Kilkenny et al., 2010). Wistar rats (*n* = 65) were obtained from the Australian Animal Resource Centre (Canning Vale, WA) and transported by air and road using Australian standard methods. Animals were housed three per box at appropriate environments in a 12 h light/dark cycle according to the standard of holding facility, with food and water provided *ad libitum*. At least 48 h habituation in the housing facility was offered before any experimental intervention. After experimentation, rats are euthanised according to ethically approved protocols.

#### 2.3.7 Freund’s complete adjuvant model of inflammatory nociception in rats

Experiments are similar to those previously performed in our lab (Morgan et al., 2017). On day 1, rats were weighed, and health checked. Baseline paw withdrawal (Randall-Selitto apparatus) and paw volume (plethysmometer) measurements were made of the left and the right hind paws. Rats were lightly anaesthetised using isoflurane inhalation, and 150 μL of Freund’s Complete Adjuvant (FCA) injected subcutaneously to the left paw pad. The right paw received no treatment. Rats were returned to their home cages and allowed to recover. On day 5, both hind paws of each rat were measured for paw withdrawal and volume (t = 0). Again, the rats were lightly anaesthetised, and the test compound (0.01–10 mg/kg, 50 μL in sterile isotonic injectable saline) was injected subcutaneously into the left affected paw pad. Rats were allowed to recover. Paw pressure measurements (force in g) to illicit a withdrawal reaction were made by Randall-Selitto methods in both hind paws at t = 15-, 30-, 60-, and 120-min post compound administration. U50488H (0.17 mg/kg) was used as a KOP receptor reference compound, morphine (0.3 mg/kg; (Perrot et al., 1999) and fentanyl (0.001–0.003 mg/kg; (Stein et al., 1988) were used as MOP receptor-active clinically relevant reference compounds. The non-selective peripherally active naloxone methiodide was used to provide insight into the peripheral *versus* central mediated effects in the *in vivo* studies in particular (Lewanowitsch and Irvine, 2002). Saline vehicle acted as a negative control. Paw volume measurements were made at t = 0 and t = 120 min using a plethysmometer (Ugo-Basile Italy). Data were analysed using a combination of Microsoft Excel and GraphPad Prism software packages.

An efficacy index (*I_e_*) was calculated to represent efficacy relative to administered dose (in molar), as normalised to a reference compound. The following formula was used;

$$Efficacy\;Index\;\left(I_{e}\right)=\left(\log\left(D*A_{t}\right)\right)/A_{o}$$

Where ***D*** is dose administered (in molar concentration), ***A***_***t***_ is the area under the curve of the test peptide, and ***A***_***o***_ is the area under the curve for the control/reference compound, morphine.

Naloxone methiodide was used in the FCA model to antagonise opioid receptor function. Naloxone was administered intraplantar [i.pl, 50 μL 1 mg/kg in saline, to the inflamed paw (Stein et al., 1988)] following the baseline measurements, under isoflurane anaesthesia 15 min prior to i.pl peptide administration. The experimental procedure for the FCA model did not vary from that described above thereafter.

#### 2.3.8 Statistics

*In vitro* assay was performed in technical duplicate or triplicate (where stated), then repeated as biological replicates. Data analysed as repeat experiments. Data is expressed as mean ± standard error of mean (SEM) unless otherwise stated. Statistical analysis was performed by Graphpad Prism software (RRID:SCR_002798). Where appropriate, *in vitro* data sets were analysed by a two-tailed, paired Student’s t-test. *In vivo* data sets were analysed by a two-way repeated measures ANOVA with Šídák’s multiple comparison test, comparing test compound-treated *versus* saline-treated. Where applicable, Kruskal-Wallis planned comparison ANOVA was applied, as indicated in the text. Significance was set at *p* < 0.05.

## 3 Results

### 3.1 Identifying potent, selective, and metabolically stable KOP receptor agonist peptides

Native Dynorphin (Dyn1-17), the smallest active fragment Dyn1-7 and the control compounds CR845 and U50488H showed a predicted high potency at the KOP receptor in our cAMP assay, with little activity in MOP receptor or DOP receptor. From the library of 46, first generation peptides, two lead peptides (Supplementary Figure S1) passed the initial cAMP high throughput screening process in HEK293-KOP receptor cells, with all others showing no reasonable activity or selectivity for KOP receptor at 1 and 10 µM. Based on the two lead peptides identified, a panel of second generation peptides were designed, synthesised, and screened for selectivity at KOP receptor, with three peptides showing low nanomolar activity.

Poor metabolic stability of these peptides required further structural refinement, which led to the third generation peptides. A large proportion of peptides showed mid-to-low range nanomolar activity in KOP receptor, often with no discernible activity below 1 µM in MOP receptor or DOP receptor, suggesting high selectivity for KOP receptor. Comprehensive peptide sequences are provided in supplementary data Table 1.

**TABLE 1:** Peptide and control compound *in vitro* properties. Activity (as EC_50_ for cAMP, nM) of each control compound tested in KOP, DOP and MOP receptor over-expressing-HEK293 cells. Stability (as half-life, t_1/2_ in minutes) tested in both trypsin and rat plasma. Compounds labelled in bold represent those with the most favourable selectivity of KOP (with little to no recordable activity in MOP or DOP) for *in vivo* testing. n.a not applicable. n.t not tested. Data shown are mean ± SEM; *n* = 3, in duplicate per data point, unless specifically indicated in parenthesis.

Compound	Activity (EC50, nM) *(n, in duplicate)*			Selectivity (fold)		Stability (t1/2, min) *(n, in duplicate)*	
	KOP	DOP	MOP	KOP/DOP	KOP/DOP	Trypsin	Plasma
Control compounds							
Dyn 1-17	3.15 (11)	>10000 (10)	3738 (10)	3175	1187	n.t	n.t
Dyn 1-7	4.3	870	3738	202	869	<1.0	8.1
U50488H	7.8 (10)	n.a	n.a	n.a	n.a	n.t	n.t
Morphine	n.a	n.a	27.3	n.a	n.a	n.t	n.t
Fentanyl	n.a	n.a	33.7	n.a	n.a	n.t	n.t
SNC80	n.a	11.7(10)	n.a	n.a	n.a	n.t	n.t
CR845	0.37	10000	10000	27027	27027	>1000	648.6
2nd generation							
KA201	1092	2176	1717	2	2	6.4	n.t
KA202	930	>10000	>10000	11	11	8.5	n.t
KA203	74.7	>10000	>10000	134	134	13.6	72
**KA204**	**7.5**	**>10000**	**>10000**	**1333**	**1333**	**15.4**	**71**
KA205	1796	3919	>10000	2	6	3259	28.7
**KA206**	**6.1**	**>10000**	**>10000**	**1639**	**1639**	**15.9**	**31.9**
KA207	6.7	>10000	3448	1493	515	7.6	38.2
3rd generation							
**KA301**	**4.54**	**>10000**	**>10000**	**2203**	**2203**	**276**	**2239.4**
KA302	11.63	1662	3997	143	344	>1000	323.9
KA303	13.5	1320	>10000	98	741	12.1	168.2
KA304	3.64	1600	363	440	100	8.33	78.99
**KA305**	**3.87**	**>10000**	**>10000**	**2584**	**2584**	**4.9**	**321**
**KA306**	**26**	**>10000**	**>10000**	**385**	**385**	**69.9**	**160.3**
KA307	1037	>10000	1866	10	2	n.t	n.t
KA308	230	>10000	5823	43	25	n.t	n.t
KA309	3.9	>10000	>10000	2564	2564	>1000	174.1
**KA310**	**1.77**	**>10000**	**>10000**	**5650**	**5650**	**>1000**	**185.7**
**KA311**	**31.05**	**>10000**	**>10000**	**322**	**322**	**>1000**	**>1000**
KA312	3.79	>10000	>10000	2639	2639	445.4	15.6
KA313	4.45	>10000	>10000	2247	2247	>1000	12.5
**KA314**	**2.49**	**>10000**	**>10000**	**4016**	**4016**	**16.1**	**18.6**

**FIGURE 1:**
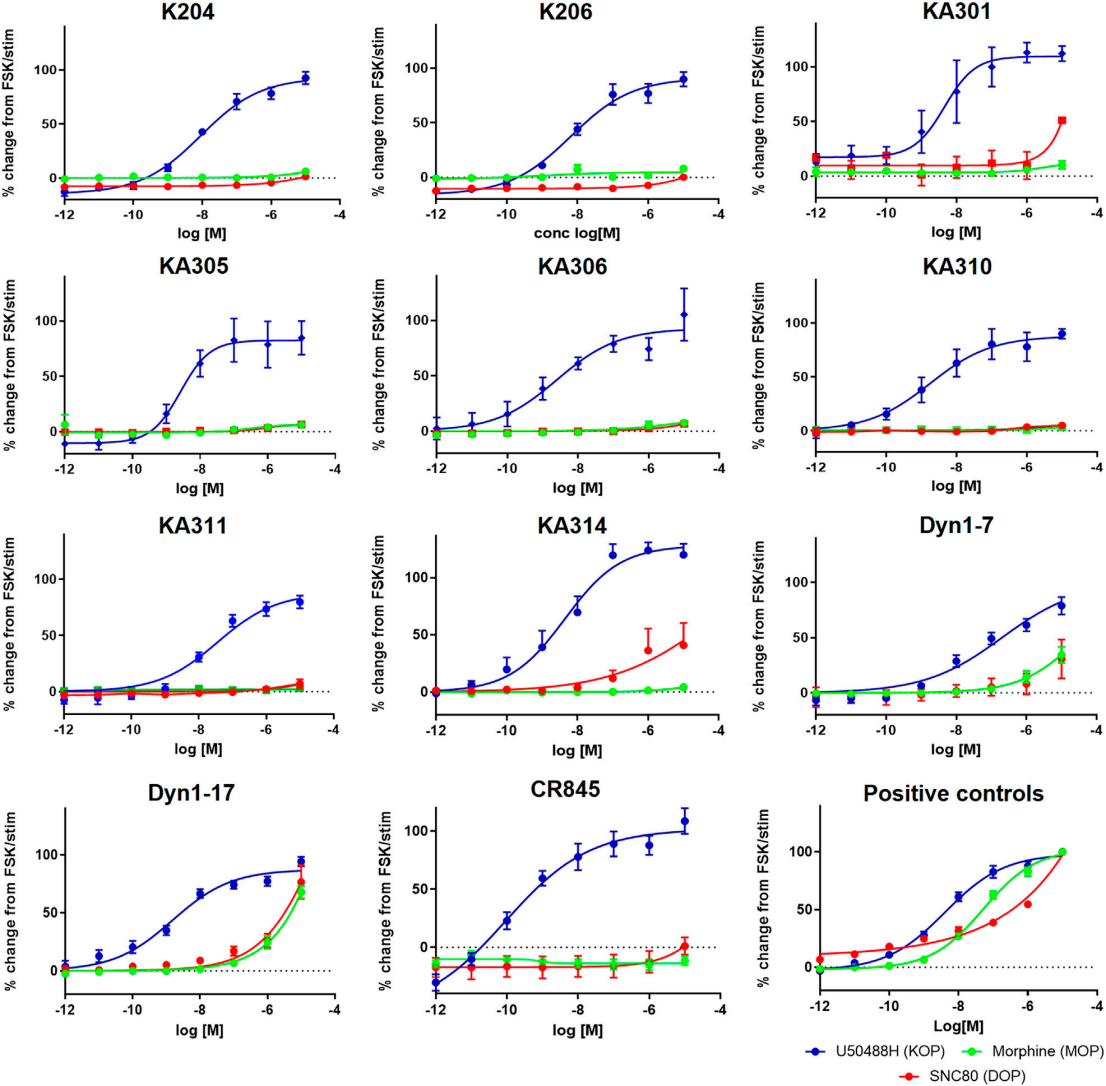
Concentration-response curves of the most promising selected peptides in the cAMP assay, compared to positive control compounds and reference peptides Dyn1-7 and Dyn1-17. HEK293-KOR cells; blue. HEK293-MOP cells; green. HEK293-DOP cells; red. *Y*-axis shows percentage change in cAMP production normalised to FSK as maximal production and clean stimulation buffer as baseline cAMP expression (0%). Data shown is an average of a minimum of three independent experiments, each in duplicates, mean ± SEM.

**FIGURE 2:**
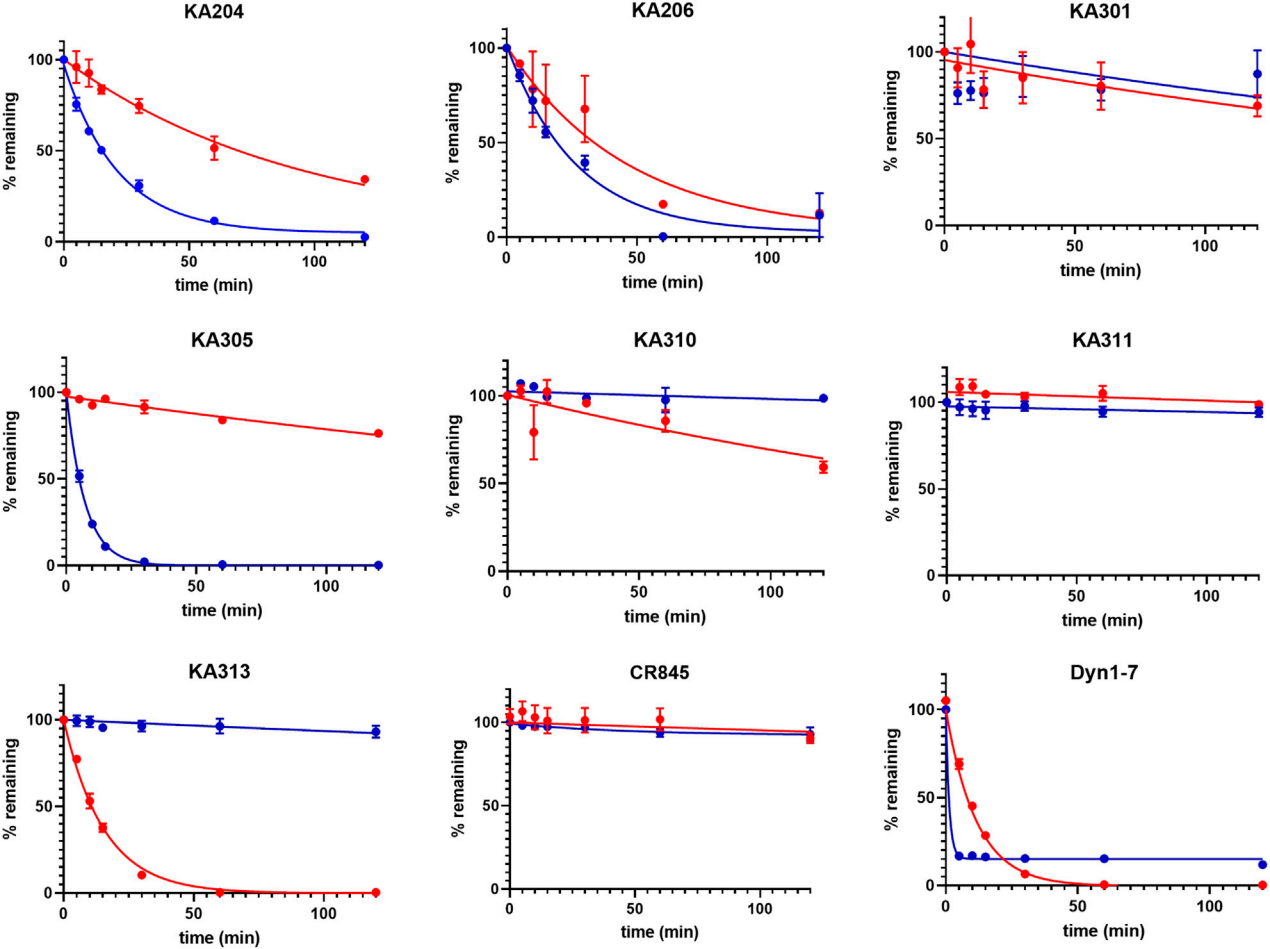
Metabolic stability of the most promising selected peptides in trypsin (blue) and rat plasma (red). The half-lives determined from these data are presented in Table 1 (means ± SEM; *n* = 3 in duplicate per data point).

**FIGURE 3:**
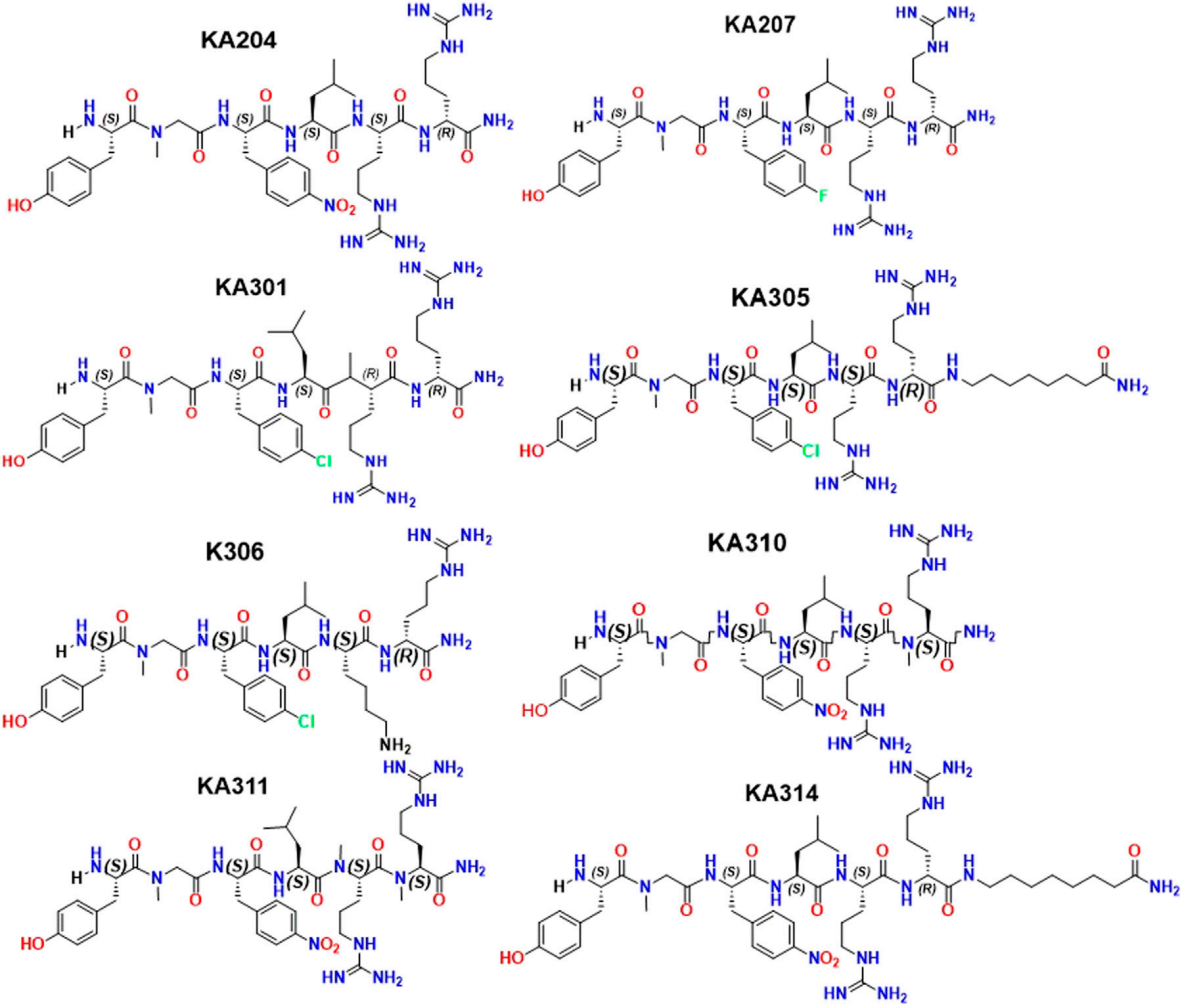
Structures of selected peptide candidates.

The most promising compounds had <50 nM EC_50_ (cAMP) at KOP receptor, a selectivity for KOP receptor over MOP receptor and DOP receptor >1000-fold (based on EC_50_), as well as a half-life >60 min in either plasma or trypsin. Figures 1–3, show concentration-response curves, stability assay data, and structures for these select compounds, respectively. With final selections for *in vivo* testing, emphasis was placed on higher plasma stability due to pharmacokinetic relevance in circulation instead of oral delivery, where trypsin has greater relevance.

Peptides were selected for further testing based on low nanomolar potencies (EC_50_ < 50 nM, cAMP assay, Table 1), a selectivity for KOP receptor >1000-fold over both MOP receptor and DOP receptor, and a half-life in rat plasma >1 h. Peptides KA204, KA305, KA310, and KA311 were selected using these criteria. The remaining most promising leads that did not meet these stringent criteria were reserved for future testing.

### 3.2 Peptide activity *in vitro* is inhibited by naloxone

**FIGURE 4:**
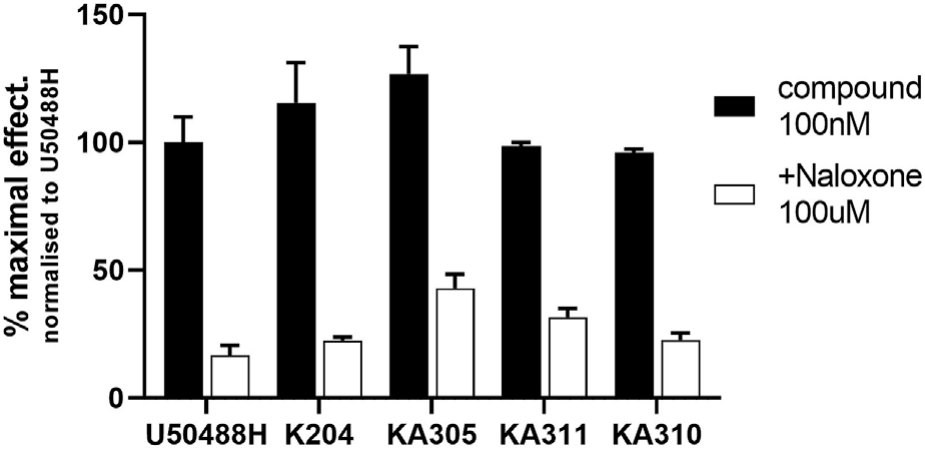
Inhibition of cAMP modulation by naloxone. Opioid antagonist naloxone (100 µM) was added to KOP receptor over-expressing HEK293 cells prior to stimulating them with lead peptide (100 nM), and assaying for cAMP activity as described. Naloxone significantly inhibited peptide activity at KOP receptors, indicating opioid-mediated receptor activity by the peptides. Mean ± SD, *n* = 3, each in duplicate. *p < 0.05 Student’s paired *t*-test from same compound in absence of naloxone.

The shortlisted peptides, KA204, KA305, KA310, and KA311 were tested for specific opioid receptor activity in HEK293-KOP receptor cells using the non-selective opioid receptor antagonist, naloxone. Naloxone significantly blocked the activity of each lead peptides tested (Figure 4), clearly indicating the activity of each peptide on cAMP production was mediated through opioid receptors and not an off-target effect.

### 3.3 Selected peptides show high binding affinity to KOP receptor

**FIGURE 5:**
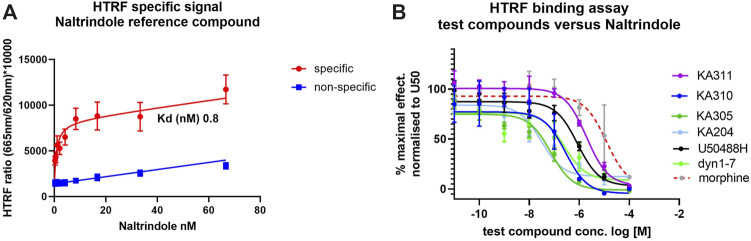
Binding affinity of test ligands to KOP receptor in a HTRF assay. **(A)** specific (red) and non-specific (blue) binding of tagged naltrindole to the KOP-expressing cells. The saturation binding coefficient (K_d_) was calculated for the reference ligand was calculated at 0.8 nM. **(B)** Completive binding assay data of test and control compounds *versus* the reference ligand (at approx. K_d.80_; 8 nM, as recommended by the manufacturer) in the HTRF assay. The IC_50_ for each compound was determined from these curves and used to calculate the K_i_ for binding to KOP in competition with naltrindole reference. Ligand (see Supplementary Table S2). *n* = 3, each in triplicate, mean ± SD.

Selected peptide candidates KA204, KA305, KA310 and KA311 along with U50488H, Dyn1-7, and Dyn1-17 were further screened for binding affinity to KOP receptor (*K*_*i*_ relative to *K*_*d*_ of naltrindole) using a homogenous time-resolved fluorescence (HTRF) assay. Morphine was used as a negative control for KOP receptor binding. The *K*_*d*_ of the reference ligand (naltrindole; as supplied in HTRF kit and recommended by the manufacturer) was determined to be 0.8 nM (*n* = 3). Test compounds were assayed for competitive binding against naltrindole (8 nM) to determine their *K*_*i*_ values (Figure 5). Calculated *K*_*i*_ values relative to naltrindole are shown in Supplementary Table S2). All test peptides assayed had *K*_*i*_ values in the low-to-mid nanomolar range comparable to Dyn1-17, Dyn1-7 control peptides and U50488H with a similar rank order as the EC_50_. As expected, morphine displayed a poor affinity for KOP receptor.

### 3.4 Involvement of KOP receptor agonist peptides in receptor desensitisation

**FIGURE 6:**
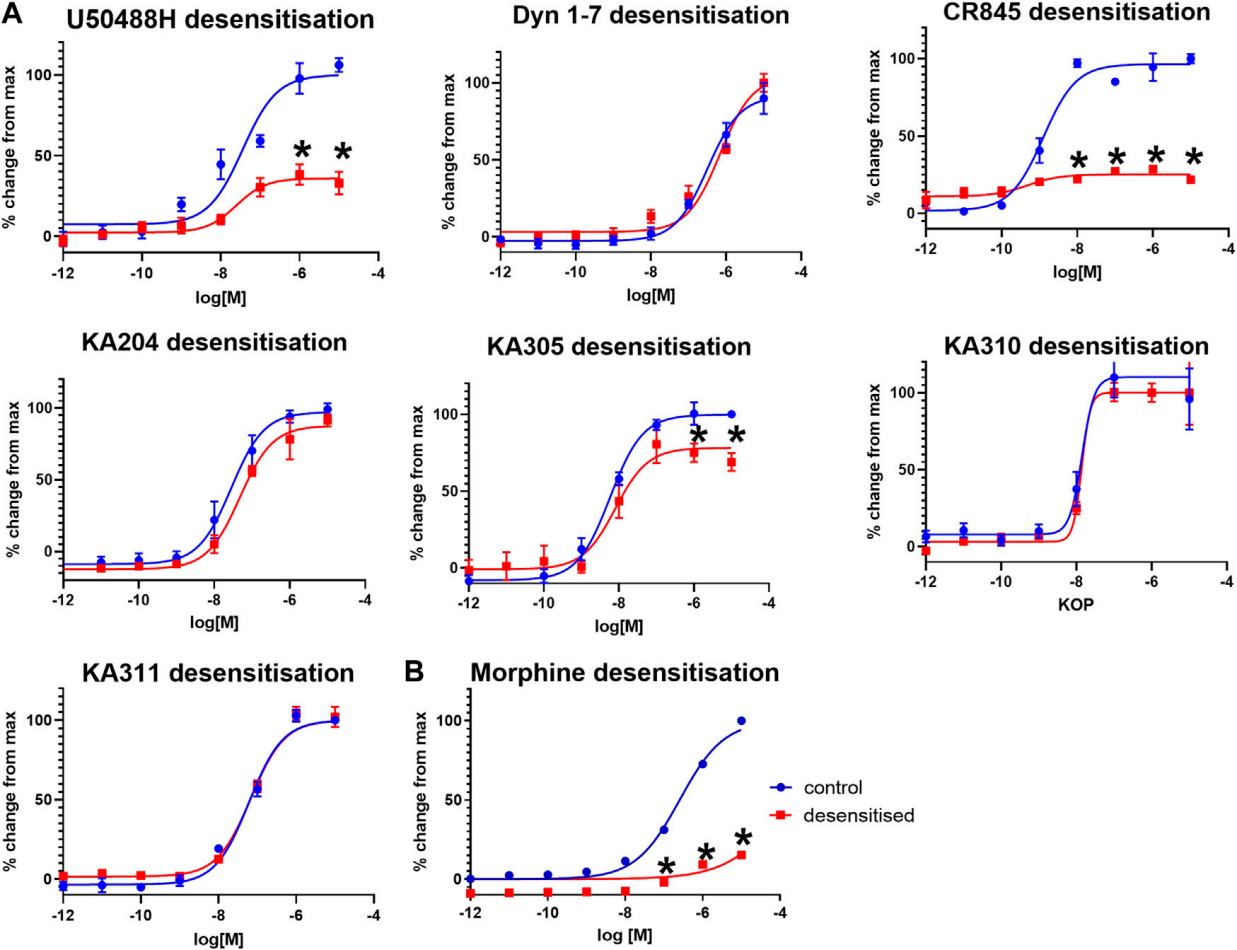
**(A)** KOP receptor desensitisation in cAMP assay in response to agonist peptides. **(B)** MOP desensitisation in cAMP assay in response to morphine. Following 6 hrs of pre-treatment with respective agonists (1 μM), KOP **(A)** and MOP **(B)** cells were restimulated with the same peptides/compound in a concentration-response manner and cAMP production measured (red), compared to non-pre-treated cells as control (blue). Mean ± SEM *n* = 3 in duplicate per data point. *p < 0.0001 unpaired two-tailed *t*-test maximal response (%change from max) compared to control.

Selected peptides were screened for KOP receptor desensitisation in HEK293-KOP receptor cells, similar to previously described (Koch et al., 2001). Pre-treatment of cells with reference compounds (1 µM) U50488H or CR845 for 6 h resulted in a significant reduction in maximal effect (cAMP % change; unpaired two-tailed *t*-test *p* < 0.0001 per) indicative of receptor desensitisation (Figure 6). Dyn1-7 showed no discernible desensitisation with a superimposable concentration-response curve with or without peptide pre-treatment. KA305 also showed modest but significant desensitisation of the test peptides (*p* < 0.005). However, the extent to which the maximal response was reduced was less than that observed for CR845 and U50488H. The candidates, KA204, KA310 and KA311, showed no indication of desensitisation, with the assay displaying identical sensitivity to the peptides even after 6 h of treatment.

As a control, morphine was tested for desensitisation in HEK293-MOP receptor cells. Morphine pre-treatment caused predictable and strong desensitisation of MOP receptor (Morgan et al., 2012) like U50488H in KOP receptor (Figure 6).

### 3.5 Bias agonism of selected peptides at KOP receptor

**FIGURE 7:**
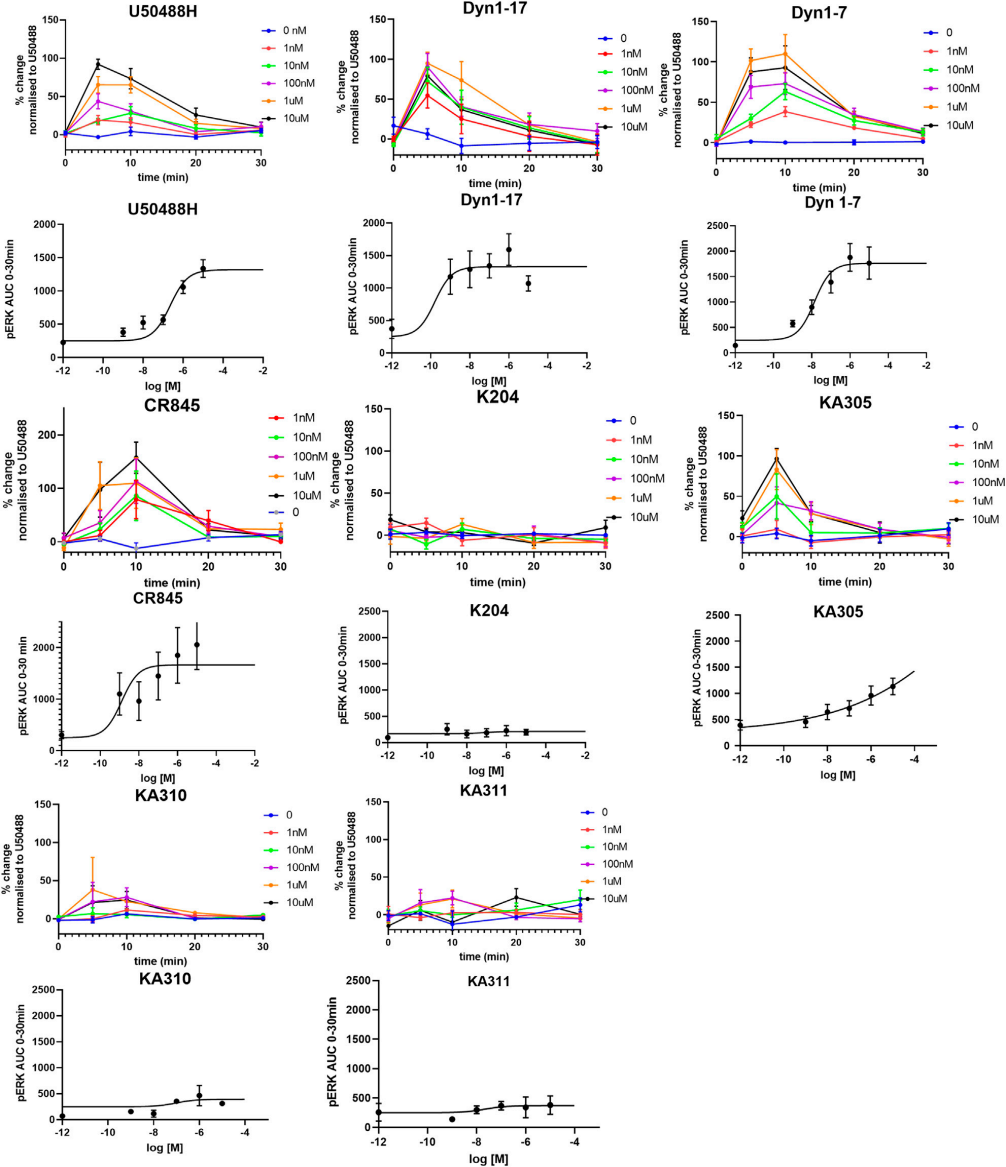
Expression of pERK_1/2_ in response to agonists in HEK293-KOP cells. For each agonist, graphs of concentration over time are plotted, from which AUC was calculated and plotted against concentration for determination of EC_50_ (sigmoidal curves). Clearly, KA204, KA310, and KA311 displayed no pERK activation. Data shown are means ± SEM; *n* = 3 in triplicate per data point.

**FIGURE 8:**
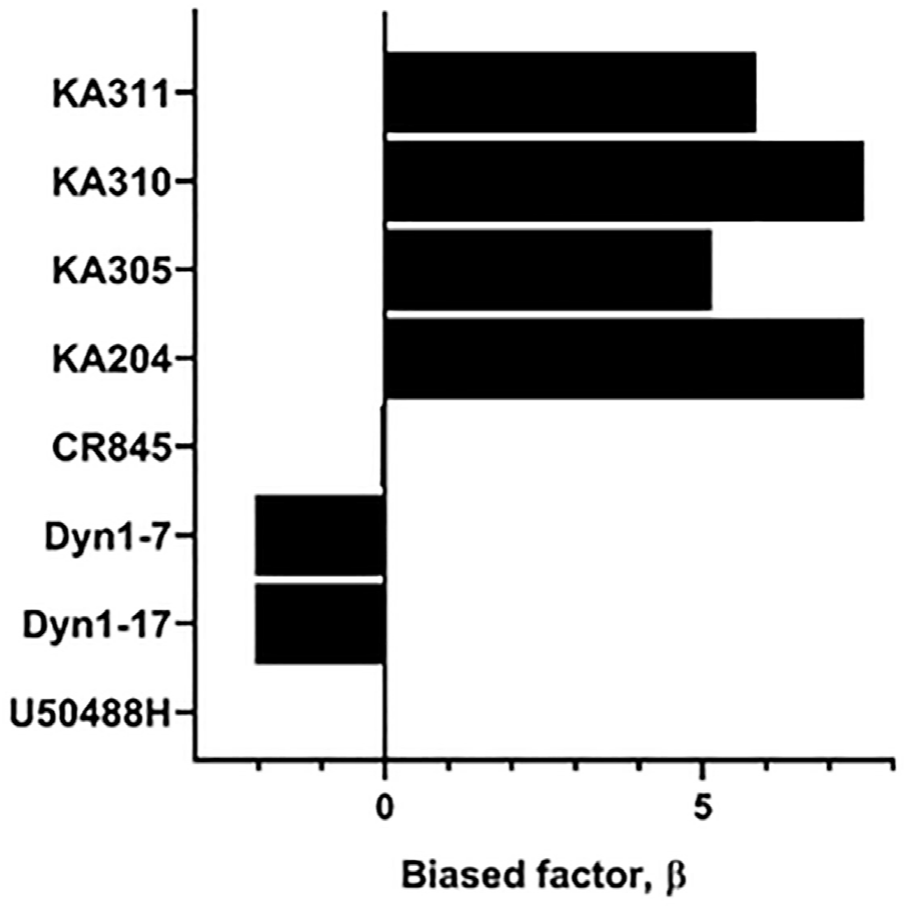
Determination of bias factor *β*, relative to U50488H, for control compounds and agonist peptides. Positive values indicate a bias towards cAMP modulation, whereas negative values indicate a bias towards pERK. U50488H by definition has a bias of zero.

Each selected peptide and control compound was tested in a quantitative pERK_1/2_ assay. U50488H, CR845, and Dyn1-7 caused time and concentration-dependent pERK expression in HEK293-KOP receptor cells. Expression of pERK was transient and short-lived, indicative of early-phase pERK release (Joo et al., 2016) (Figure 7). KA305 influenced pERK induction at higher concentrations, lacking the classical sigmoidal dose-response curve. KA204, KA310, and KA311 did not show any pERK activity. A bias agonist factor *β* was calculated (Rajagopal et al., 2011) (Figure 8) for each peptide/compound, using U50488H as a reference compound; negative *β* values indicate a bias for pERK activation (left shift), and positive *β* values (right shift) indicate a bias for cAMP modulation relative to U50488H, respectively. The lack of pERK activity predicted that KA204, KA305, KA310, and KA311 preferred cAMP, in a similar manner to that observed for U50488H Interestingly, KA305, which showed pERK activity at higher concentrations, showed a *β* value indicative of bias towards cAMP. Dyn1-7 showed bias towards pERK, whereas CR845 had no bias either way, showing identical signal preference to U50488H.

### 3.6 Antinociceptive efficacy in the FCA model of inflammatory hyperalgesia

**FIGURE 9:**
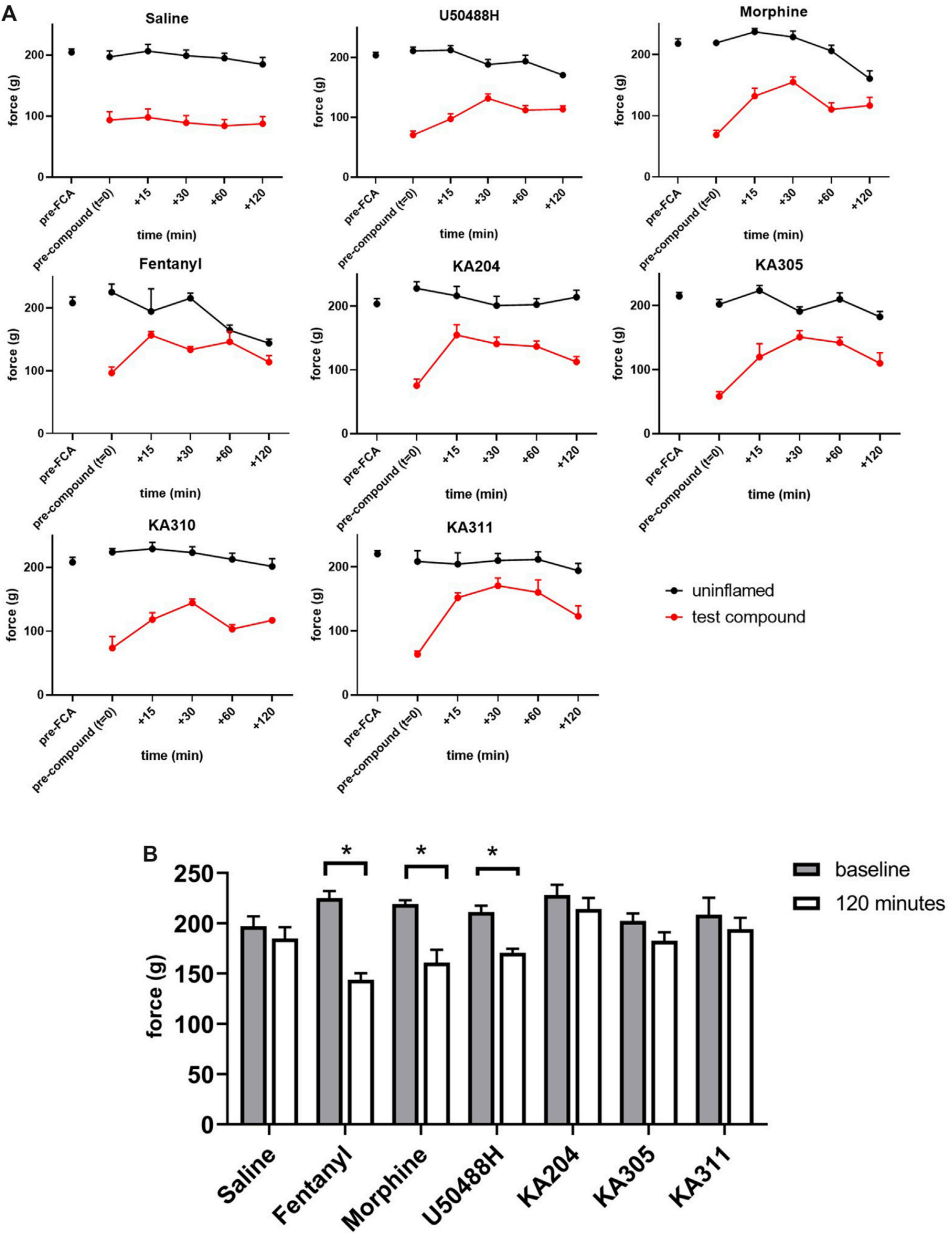
Threshold of paw withdrawal to mechanical stimulus, over time as measured by Randall-Selitto assay, in the FCA model of inflammatory pain. **(A)** Force required to elicit a paw-withdrawal response for individual compounds tested in the FCA-treated paw and un-inflamed paw (contralateral), after compound administration (after *t* = 0). Data shown are means ± SEM; *n* = 6 per group. *p < 0.05, significant difference between *t* = 0 and *t* = 120 min in contralateral paw **(B)** Force required to elicit a paw withdrawal response in the contralateral/un-inflamed paw at baseline (*t* = 0, prior to compound administration) and at *t* = 120 min following compound administration in the ipsilateral paw. Data shown are means ± SEM; *n* = 6 per group. *p < 0.05, significantly different as indicated; paired *t*-test.

Compounds KA204, KA305, KA310, and KA311 all proved to have relevant drug-like attributes with respect to low nanomolar potency, high selectivity for KOP receptor over other opioid receptors, as well as favourable metabolic stability in trypsin and plasma. Consequently, these peptides were selected for *in vivo* testing in the Freund’s Complete Adjuvant (FCA) model of inflammatory pain (Iwaszkiewicz et al., 2013; Morgan et al., 2017). Administration of KOP receptor agonist U50488H (0.17 mg/kg i. pl) directly to the inflamed paw of the rat caused an increase in paw withdrawal threshold (in grams) closer to baseline levels, characteristic of an opioid-like antinociceptive effect (Figure 9). This effect was short-lived (<60 min), returning to the original withdrawal threshold before compound administration by 120 min. These data validate that the local administration of KOP receptor agonists has antinociceptive effects. KA311, with its superior metabolic stability profile (*c.f.* KA204, KA305, KA310) was selected as a model compound for *in vivo* dose-response curve generation (Supplementary Figure S2), whereby local i.pl administration of 0.01, 0.1, 0.3, 5, and 10 mg/kg peptide to the inflamed paw was tested for analgesic efficacy to determine the maximal possible effect (MPE). The area under the curve for the force for withdrawal *versus* time curves (Supplementary Figure S3) was plotted against the dose to generate a dose-response curve (Supplementary Figure S2).

From this curve, the dose closest to the 80th percentile of maximal possible effect (MPE_80_) was determined to be 0.3 mg/kg. Not all peptides were tested in such a dose-response manner *in vivo*, in adherence to the 3 Rs of animal research (Guhad, 2005; Burden et al., 2015). However, this dose of 0.3 mg/kg was subsequently used for preliminary *in vivo* screening for all peptides, an assumption based on the similar *in vitro* potencies, binding, stability and backbone structure of each respective peptide. KA204, KA305, KA311 (0.3 mg/kg, i.pl *n* = 6 each). All showed antinociceptive activity (Figure 9). All compounds showed significantly improved paw withdrawal threshold without effecting oedema (not shown) at most time points post administration (*p* > 0.05, repeated measured ANOVA compared to saline, Figure 9). KA310 proved ineffective at modulating nociception (not shown) and was therefore discontinued from further testing. Since there was no effect on oedema/swelling of the affected paw at the administered dose, the observed antinociceptive effect was not due to an anti-inflammatory event, but an opioid-like analgesic effect.

Interestingly, all three reference compounds, morphine, fentanyl and U50488H, showed effects in the contralateral (uninflamed and untreated) control paw by 120 min following its administration into the ipsilateral paw (Figure 9), whereby the paw withdrawal threshold was significantly reduced compared to baseline (time point t = 0, *p* > 0.05 paired *t*-test). These apparent acute allodynia-inducing effects were not observed for saline control or our test peptides.

The area under the curve (AUC) of each compounds’ *in vivo* effect was calculated as a surrogate estimate measure for overall efficacy (Supplementary Figure S3A). Individual peptides were compared to control compounds (U50488H, morphine (0.3 mg/kg), and fentanyl (3.3 μg/kg)). KA311, KA204, and KA305 (each 0.3 mg/kg, 2.0 mM, 2.07 mM and 1.8 mM, respectively) showed significantly better efficacy than U50488H.

An Efficacy Index (*I_e_*) was calculated for each peptide/compound, giving a relative effectiveness score per unit of molar dose (Supplementary Figure S3A). Fentanyl showed a very high *I_e_* simply due to the exceptionally low dose administered (1.0 μg/kg). KA204, KA305, and KA311 showed statistically significant higher *I_e_* over morphine (Kruskal-Wallis ANOVA; morphine is reference; therefore, *I_e_* is 0). KA305 and KA311, which showed the most promising improved *I_e_* over morphine, were tested against naloxone for opioid-receptor specificity in the FCA model.

**FIGURE 10:**
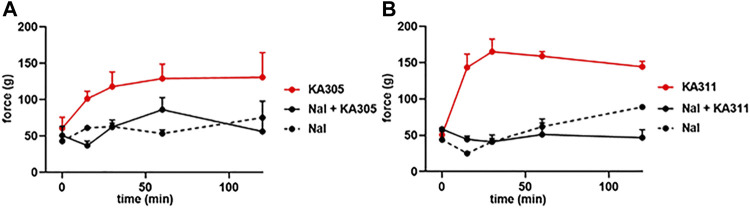
Effect of naloxone (1 mg/kg) on peptide-induced antinociception in the FCA model for **(A)** KA305 0.3 mg/kg. **(B)** KA311 0.3 mg/kg. Note the effect of each peptide is blocked by pre-administration of naloxone. Data shown are means ± SEM; *n* = 3 per group.

Naloxone pre-treatment effectively blocked the activity of each peptide, clearly indicating the antinociceptive effects were opioid receptor-mediated (Figure 10). A selective KOP antagonist (i.e., norBNI, JDTic, or CERC501) would clarify whether the effects were *via* KOP receptor and not MOP receptor or DOP receptor. However, earlier *in vitro* experiments in transfected cell lines clearly showed negligible activity of these peptides at MOP receptor or DOP receptor at reasonable concentrations. Collectively, this data provided confidence the antinociceptive effects of the selected peptides observed in the FCA model of inflammatory pain were likely elicited by KOP receptor.

## 4 Discussion and conclusion

To contribute towards tackling the opioid crisis, we interrogated how rationally designed structural modifications of Dyn1-7, the smallest active fragment of the endogenous KOP receptor peptide dynorphin, impacted potency and selectivity at KOP receptor, the metabolic stability and overall druggability of the peptides. We aimed to develop structurally stable, potent KOP receptor selective, bias agonist peptides that are potentially safer to use than current MOP receptor clinically relevant compounds.

In this work, a library of Dyn1-7 peptide analogues was rationally designed to demonstrate improved metabolic stability whilst maintaining or even improving the functionality (efficacy and selectivity over KOP receptor) of each peptide over Dyn1-7. Using cAMP modulation, a surrogate measure for the analgesic properties of opioids in cell-based assays, we identified four lead peptides (KA204, KA305, KA310, KA311), all of which showed low nanomolar potency for cAMP modulation in HEK293-KOP cells and >1000-fold selectivity for KOP receptor, over MOP receptor and DOP receptor. In standard *in vitro* stability assays, each peptide had a metabolic t_1/2_ in plasma of >60 min and improved trypsin stability, which were indefinite (>1,000 min) for KA310 and KA311. Further, our shortlisted peptides KA204, KA305, and KA311 proved to be strong binders to KOP receptor, each having low nanomolar *K*_*i*_ values as determined in an HTRF assay.

Desensitisation has been linked to receptor/G-protein uncoupling and internalisation events (Kelly et al., 2008; Rajagopal and Shenoy, 2018). The activation of MAP kinases [such as phosphorylated extracellular regulated kinase 1 and 2 (pERK_1/2_)] by G-protein coupling are often linked to β-arrestin recruitment (Thompson et al., 2015), receptor internalisation (Borgland, 2001), recycling and desensitisation (Al-Hasani and Bruchas, 2011). Furthermore, many authors have speculated an involvement for MAPKs like pERK_1/2_ in developing tolerance (Wang et al., 2009; Rodrigues et al., 2012; Joo et al., 2016). Thus, lead peptides were screened for acute desensitisation and ERK phosphorylation, a potential contributor to desensitisation and tolerance (Camilleri, 2008; Hughes et al., 2013; DiMattio et al., 2015). Similar to previous reports (Borgland, 2001), we found Dyn1-7 did not induce desensitisation, nor did KA204, KA310, and KA311. Dyn1-7 is thought to lack many of the side effects of the MOP receptor and KOP receptor clinical/experimental small molecules, especially tolerance, which may relate to the lack of desensitisation in this context.

Certainly, desensitisation-related acute or long-term reduction of receptor expression on the cell surface following internalisation impairs cell responsiveness to any given drug, and therefore, possibly leading to physical tolerance. Yet, the opposite has also been suggested, whereby desensitisation may be an adaptive means to avoid tolerance by reducing receptor-mediated signalling (Borgland, 2001). This may be the case for morphine, which induces desensitisation *via* means other than internalisation (Koch et al., 2001; Koch and Hollt, 2008). Conversely, Dyn1-7 does not cause desensitisation but does promote internalisation (DiMattio et al., 2015). Indeed, an endogenous ligand like dynorphin is unlikely to induce tolerance since it would have failed early in the evolutionary process, dictated by its intrinsic metabolic instability. Hence, internalisation and desensitisation correlations are likely to be compound-specific. With the apparent exception of Dyn 1-7, analogues KA204, KA310, and KA311 that did not show desensitisation also showed a lack of pERK activity, giving them a notable signalling bias in favour of cAMP (bias factor *β* > 5, Supplementary Table S2). Likewise, U50488H (Kaneko et al., 1994) and CR845 showed distinct desensitisation and potent ERK phosphorylation with generally no signalling bias towards cAMP. KA305 appeared to fall somewhere in between; however, it displayed significant desensitisation. The extent of reduced maximal signalling was vastly less than that observed in U50488H. It showed pERK activation at only higher concentrations, resulting in a signalling bias *β* towards cAMP. Perhaps there are, in certain circumstances (as is apparent for Dyn1-7) correlations between pERK and desensitisation. It is intriguing to us why Dyn1-7 and KA305 show pERK activity but not the analogues KA204, KA310, KA311. When scrutinising the structural chemistry of KA305, the phenylalanine residue is replaced with a *para-chloro* phenylalanine in the message region, while also possessing a *C*-terminal 2-amino heptanoic acid ‘cap’ in the address region. In contrast, the phenylalanine residues of KA204, KA310, and KA311 have a *para-nitro* substitution, which could plausibly dictate whether desensitisation is featured. Future studies would be needed to investigate how such a signalling bias of KA204, KA310 and KA311 affects other pathways such as MAPKs like p38, arrestins and calcium, and how these in turn affect cell responses. The bias factor presented is merely intended as a snapshot of efficacy of the ligands activating one pathway over the other, and should only be taken as an indicator of signalling bias, rather than finite fold-differences of potency at one intracellular pathway over the other compared to the reference ligand. It also should be noted that these signalling biases may be cell-specific, and future research should scrutinise the activities of these peptides in different native cell types that express KOP receptor, such as SH-5Y neuroblastoma cells (Cheng et al., 1995) or primary DRG neurons (Moy et al., 2020).

All the lead compounds excluding KA310 showed significant antinociceptive activity *in vivo*, which, as is canonical, is likely related to cAMP signalling due to the lack of pERK activity by these peptides. KA311 and KA305 especially were shown to temporarily alleviate the inflammatory hyperalgesia induced by FCA administration in rats with an efficacy comparable to the clinical analgesic morphine, and the reference KOP agonist U50488H. Thus, not only has this work identified lead molecules that are active *in vivo*, it verifies that the KOP receptor is a valid target for peripheral analgesia. Even though the recorded antinociceptive effect lasts no longer than 120 min, we have administered the compound directly to a highly inflamed tissue region. This inflamed tissue has an abundance of enzymes not typically expressed in (uninflamed) tissue. Since we administer it directly to that site, the more expeditious metabolism of the native linear peptide by these inflammatory enzymes is expected (Morgan et al., 2017). Also, the inflamed tissue is excessively oedematous, so it is expected to have high lymphatic drainage promoted by physical and osmotic pressures in the tissue, which will also expedite clearance of the locally injected peptide. Therefore, even though the effect may appear relatively short-lived (2 h) after local administration, such durational limitations are expected given the nature of the model. It should also be pointed out that neither morphine, fentanyl, nor U50488H provided for any longer-term efficacy over the peptides, highlighting the tissue’s nature and the limited duration of action following a local administration even with the most common clinical agent. Systemic administration (i.v, s.c.) may be anticipated to give longer-term efficacy, and while the oral route is also worth investigating, these aspects are outside the scope of the current research and will be considered for future experiments.

An interesting finding was morphine, fentanyl, and U50488H’s acute effects on contralateral paw nociception. After 2 h, the contralateral paw showed a significant allodynic-like hyper-sensitivity to these control compounds only. Acute hypersensitivity to both mechanical and thermal stimuli following morphine treatment has been previously described in neonatal rats as spontaneous withdrawal (Sweitzer et al., 2004). It is plausible these effects are due to the spinal influence of morphine on contralateral sensitivity, where morphine exposure may influence an increase in ventral-root reflex excitability, manifested as allodynia and hyperalgesia, which may involve Aδ, Aβ and c-fibres (Sweitzer et al., 2004). Desensitisation of receptors in spinal/DRG neurons may be involved in this hypersensitivity phenomenon (Bohn et al., 2002). However, considering these effects were not observed in KA204, KA311 or KA305, they may not relate to pERK *per se*. The lack of this hypersensitivity in the peptides may indicate a lack of BBB penetration or pharmacokinetic limitations of the lead peptides *versus* the small molecules, whereby the spinal and central circuits are not affected by the peptides. Indeed, direct brain permeability measurements would help clarify this, although, considering the physicochemical properties of the peptides, it is unlikely they possess any BBB permeability (Banks, 2015).

Future research will need to delineate the impact these peptides may have on tolerance, addiction and centrally-mediated acute effects on behaviour using rigorous and well-defined behavioural assays such as the open field test, rotarod test, elevated plus maze, place preference and repeated dosing studies.

Overall, the results presented are highly encouraging and provide clear evidence that a rational design approach effectively yielded stable, selective, biased KOP receptor agonist peptides based on the native Dynorphin structure. The shortlisted peptides KA311 and KA305 are highly potent KOP receptor, selective agonists, with efficacy *in vivo* that compares to morphine, both in a relative dose (i.e., 0.3 mg/kg) and in an efficacy index comparison.

The outcome from work carried out in this program presents a crucial step in the opioid pain management field. Given its promise, our work warrants a host of follow-up studies to fully maximise the potential of these lead peptides for clinical use. Further optimisation would provide confidence that such peptides will be exceptionally valuable in the clinic, providing safer and potentially less addictive agents to tackle the growing issues of the opioid crisis revolving around opioid use, misuse, and chronic pain. In doing so, we anticipate providing an improved quality of life for chronic pain sufferers.

## Data Availability

The original contributions presented in the study are included in the article/Supplementary Material, further inquiries can be directed to the corresponding author.
