# Supplementary material for: Design and development of novel, short, stable dynorphin-based opioid agonists for safer analgesic therapy
Source: Front Pharmacol. 2023 Mar 3;14:1150313. doi: 10.3389/fphar.2023.1150313 (PMC10020352; doi:10.3389/fphar.2023.1150313)
Supplement: Supplementary file 1 [file DataSheet1.pdf]

Supplementary Material

Design and development of novel, short, stable dynorphin-based opioid agonists for safer analgesic therapy

Dr Rink-Jan Lohman*, Dr Karnaker Reddy Tupally*, Dr Ajit Kandale*, A/Prof. Peter J. Cabot# and Dr Harendra S Parekh#

# Correspondence:
Associate Professor Peter J. Cabot, B.App.Sci. PhD.
School of Pharmacy
The University of Queensland
Pharmacy Australia Centre of Excellence
20 Cornwall Street
Woolloongabba QLD 4102 Australia
Email:p.cabot@uq.edu.au

Dr Harendra S Parekh PhD. BScPharm. MRPharmS.
School of Pharmacy
The University of Queensland
Pharmacy Australia Centre of Excellence
20 Cornwall Street
Woolloongabba QLD 4102 Australia
Email:h.parekh@uq.edu.au

1 Supplementary Figures and Tables

1.1 Supplementary Figures

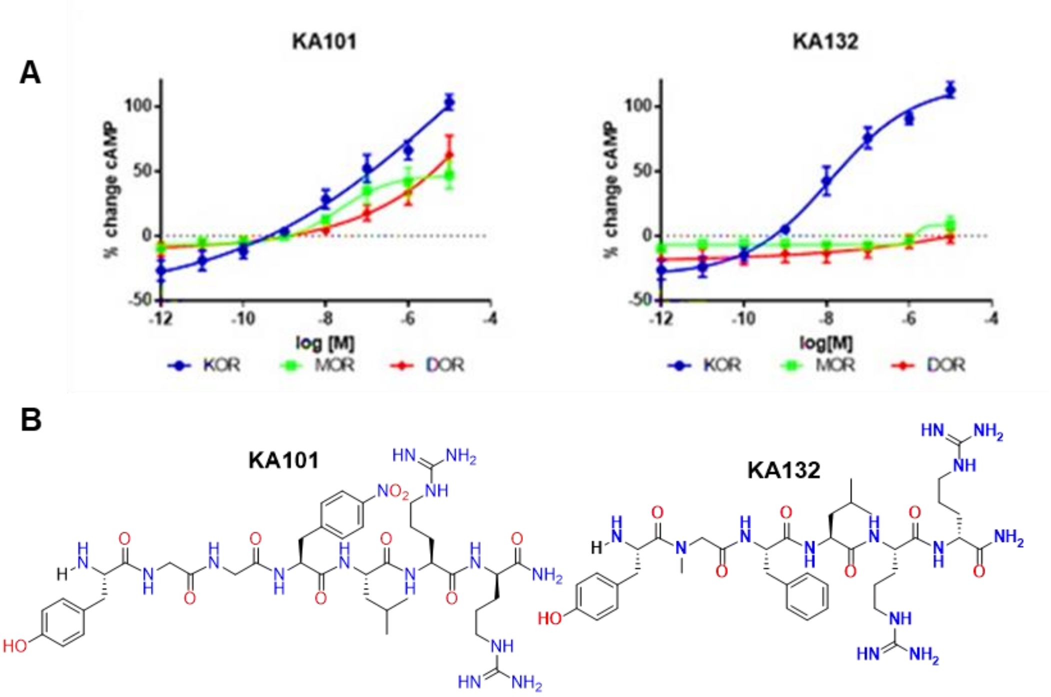

C

								Activity (EC50, nM)			Stability (t1/2,mins)	
	sequences (*N to C*)											
Peptide	aa1	aa2	aa3	aa4	aa5	aa6	aa7	KOP	DOP	MOP	Trypsin	Plasma
KA101	Tyr	Gly	Gly	4NO2-Phe	Leu	Arg	Arg	14.4	521.8	187.4	<0.1	8.3*
KA132	Tyr	Sar	Phe	Leu	Arg	Arg	---	8.6	>10000	6700	1.8	96.7*

Supplementary Figure 1. *A; Concentration-response curves of the KA101 and KA 132 in the cAMP assay. B; Structures of KA101 and KA132 C; sequences for peptide KA101 and KA132 and respective EC50 (nM) at*

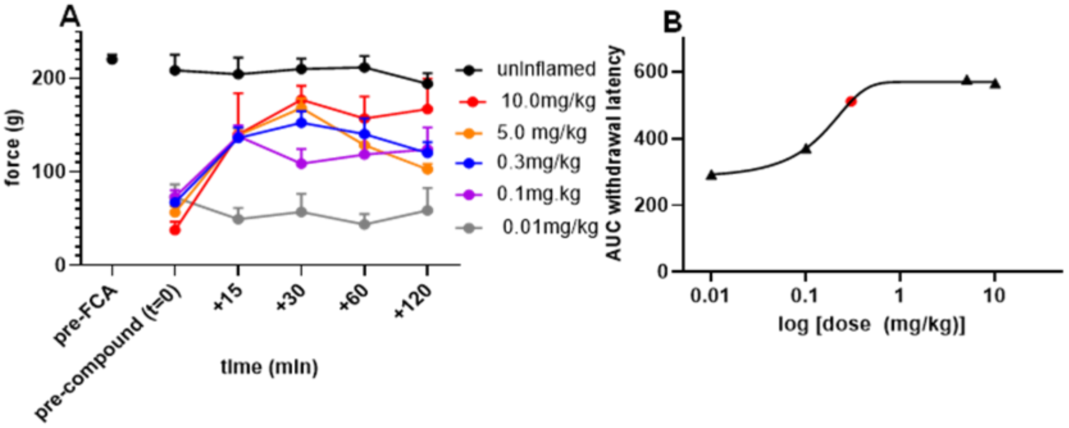

Supplementary Figure 2. *In vivo dose-response of KA311 in the FCA-model of inflammatory pain. A: Analgaesiometry/paw withdrawal threshold of KA311 measured by Randal-Selitto assay in the Freund's Complete Adjuvant (FCA) rat model of inflammatory pain. Shows grams of force required to elicit a paw-withdrawal response before inflammation (pre-FCA), before compound administration on day 5 (pre-compound, t=0) and at time points (in minutes) after KA311 administration at different doses. B; Dose-response curve of KA 311. Graph derived from the area under curve (AUC) from the time/force data obtained in the analgaesiometry assay for multiple doses of KA311 compared to baseline (saline only). Red data point denotes the MPE80 (at 0.3mg/kg). n=3-6 per dose point.*

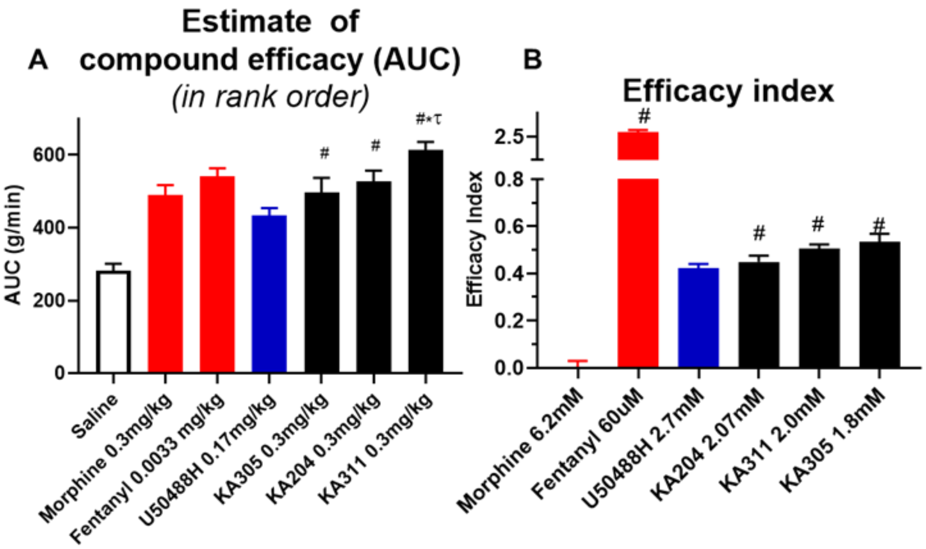

Supplementary Figure 3. *A: Estimate of compound efficacy, demonstrated as area under curve (AUC, g/min), for each peptide in the FCA inflammatory pain model. Mann-Whitney T-test planned comparisons show significance of peptide versus control compounds. # p<0.05 vs U50488H, * p<0.05 vs morphine, τ P<0.05 vs fentanyl. Mean ±SEM. B: Efficacy index (I_e) of test peptides versus control compounds, showing relative efficacy per unit dose (molar) in the FCA model of inflammatory pain in rats. Mean±SEM, # p<0.05 versus morphine planned comparison, Kruskal-Wallis ANOVA.*

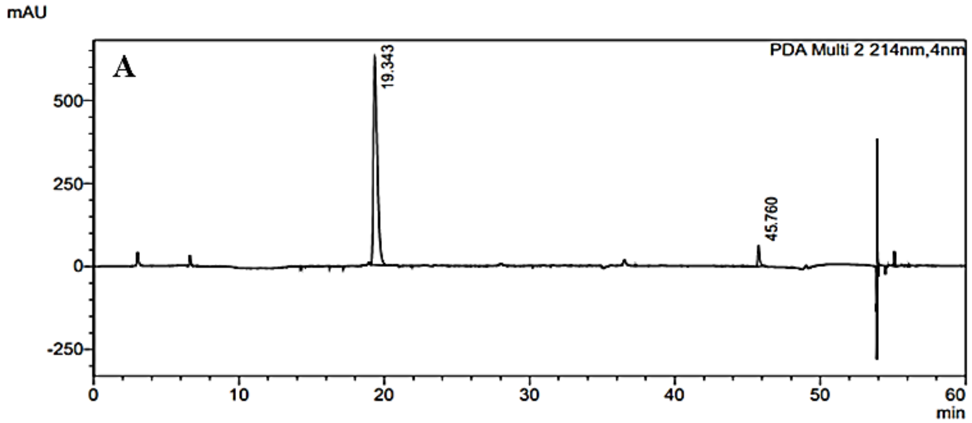

<Peak Table>

PDA Ch2 214nm

Peak#	Ret. Time	Area	Area%
1	19.343	11363259	95.388
2	45.760	549403	4.612
Total		11912662	100.000

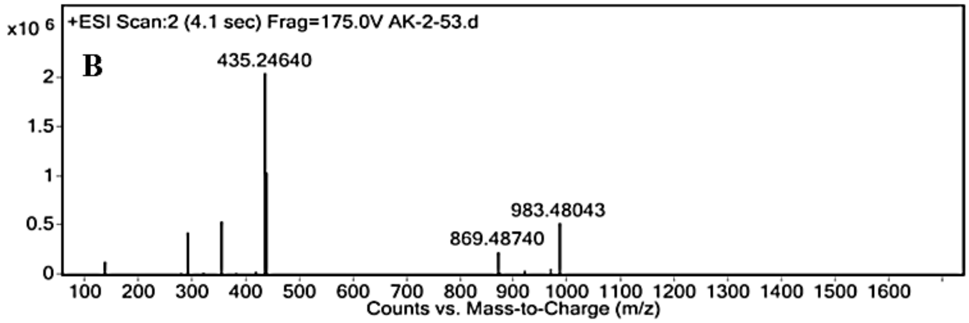

Supplementary Figure 4. *HPLC(A) and HRMS (B) spectra of KA204*

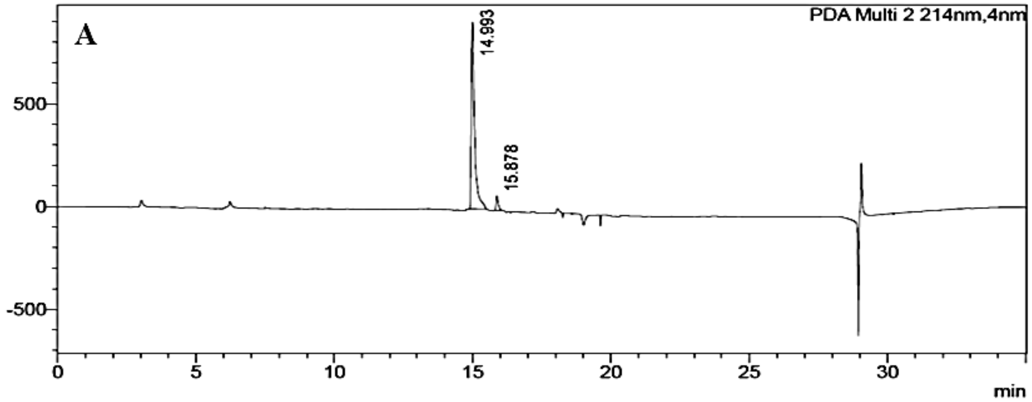

<Peak Table>

PDA Ch2 214nm

Peak#	Ret. Time	Area	Area%
1	14.993	7415320	94.929
2	15.878	396100	5.071
Total		7811420	100.000

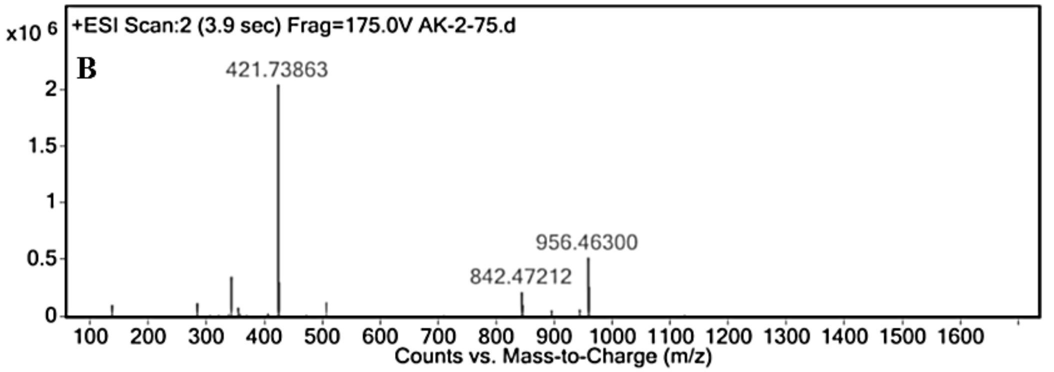

Supplementary Figure 5. *HPLC(A) and HRMS (B) spectra of KA207*

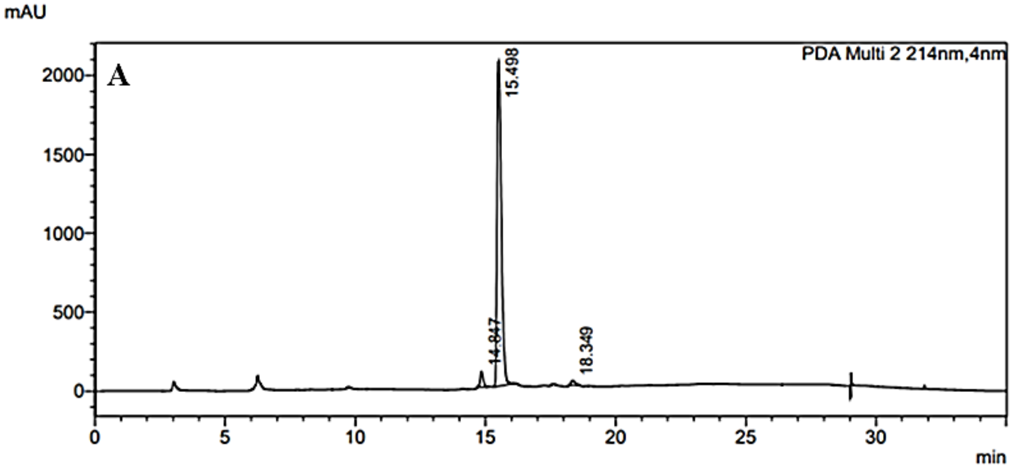

<Peak Table>

PDA Ch2 214nm

Ret. Time	Area	Area%
14.847	783081	3.185
15.498	23488728	95.525
18.349	317402	1.291
	24589212	100.000

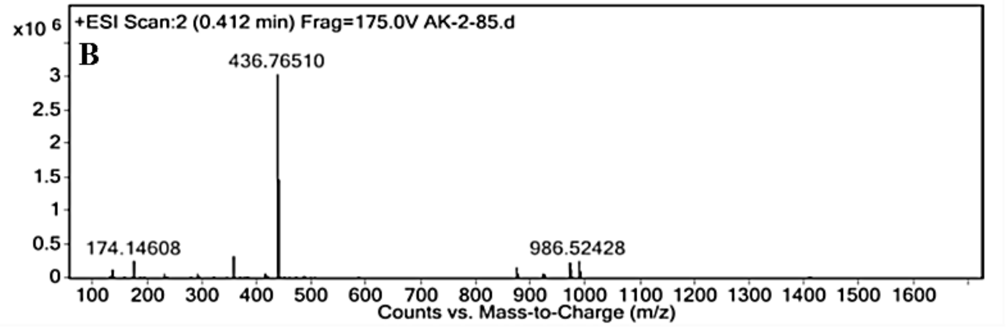

***Supplementary Figure 6**. HPLC(A) and HRMS (B) spectra of KA301*

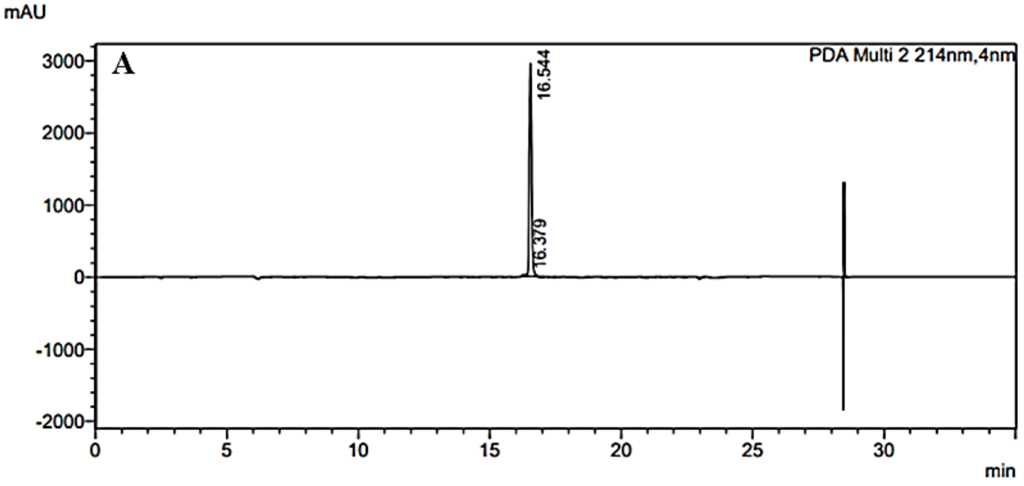

<Peak Table>

PDA Ch2 214nm

Ret. Time	Area	Area%
16.379	65638	0.368
16.544	17759225	99.632
	17824863	100.000

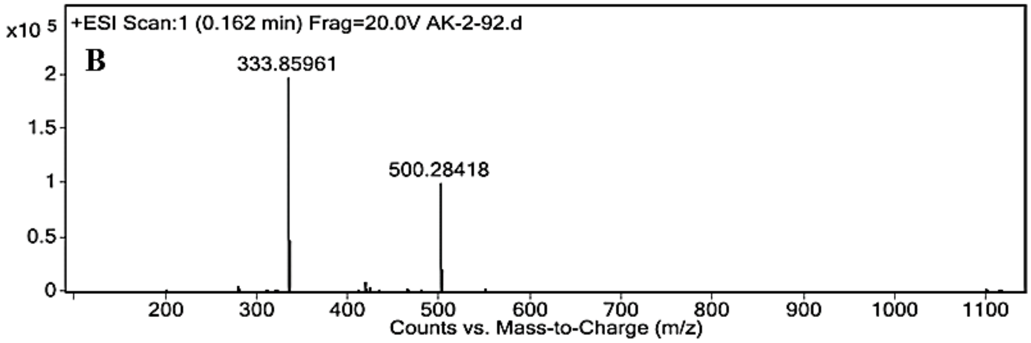

***Supplementary Figure 7**. HPLC(A) and HRMS (B) spectra of KA305*

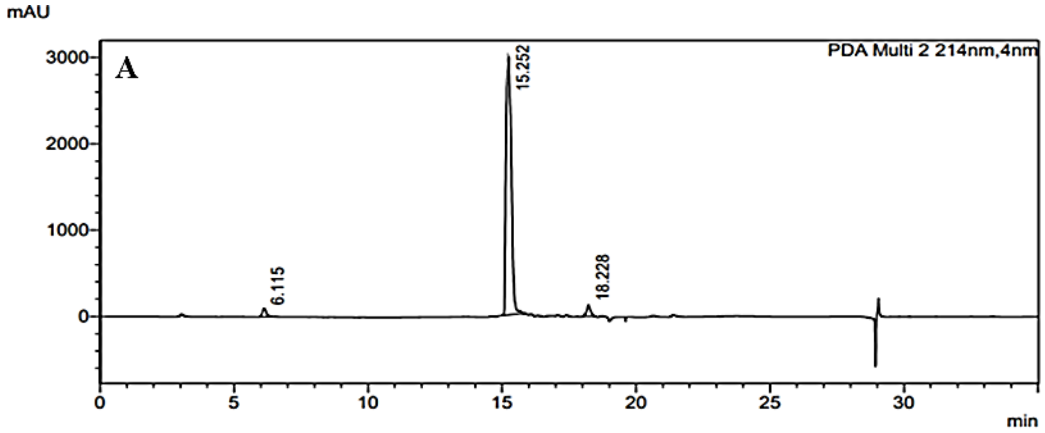

<Peak Table>

PDA Ch2 214nm

Ret. Time	Area	Area%
6.115	931002	2.139
15.252	41322940	94.925
18.228	1278125	2.936
	43532067	100.000

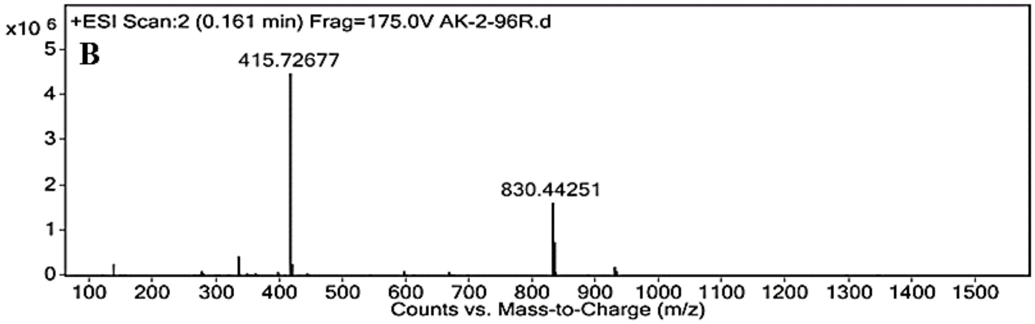

***Supplementary Figure 8**. HPLC(A) and HRMS (B) spectra of KA307*

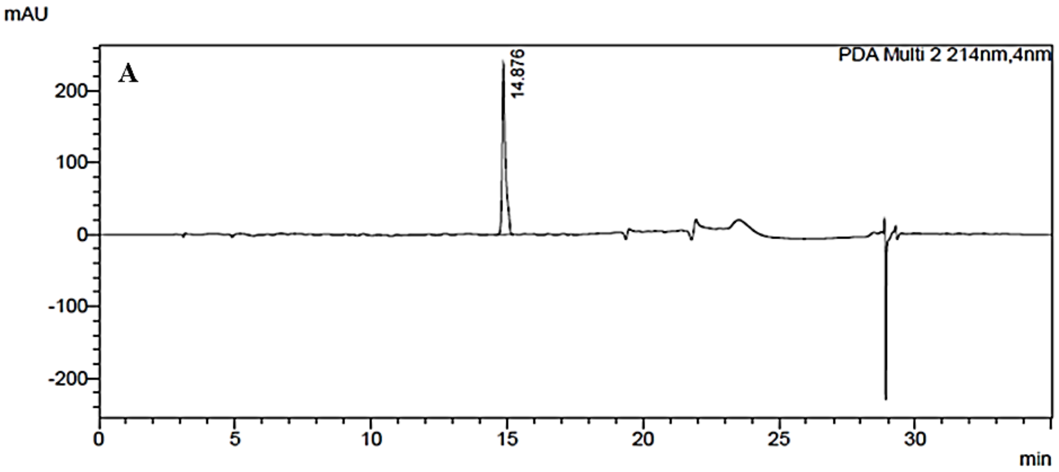

<Peak Table>

PDA Ch2 214nm

Peak#	Ret. Time	Area%
1	14.876	100.000
Total		100.000

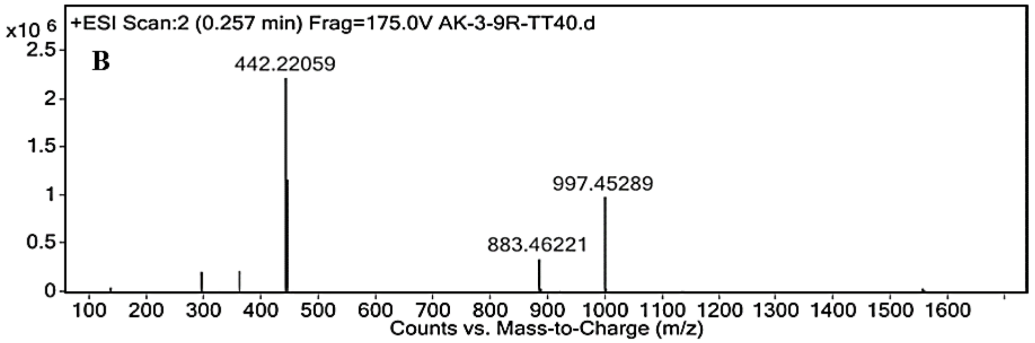

***Supplementary Figure 9**. HPLC(A) and HRMS (B) spectra of KA308*

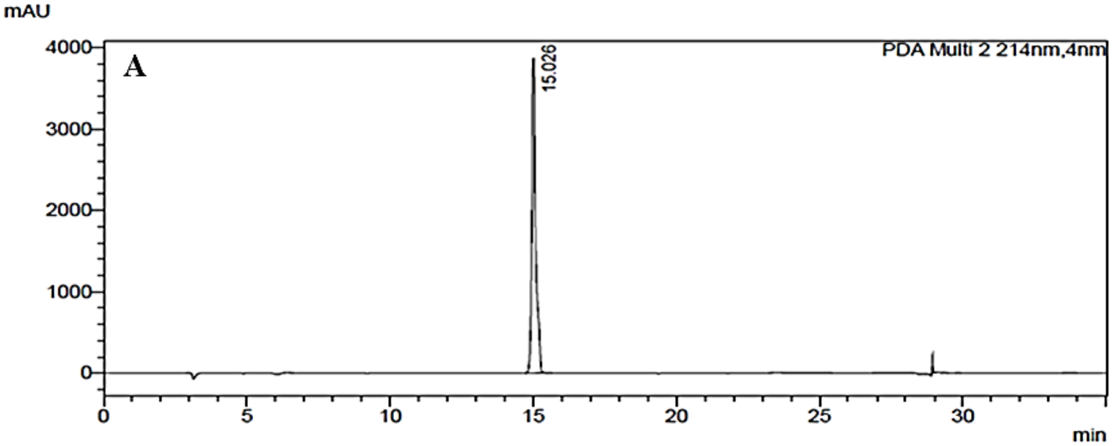

<Peak Table>

PDA Ch2 214nm

Peak#	Ret. Time	Area%
1	15.026	100.000
Total		100.000

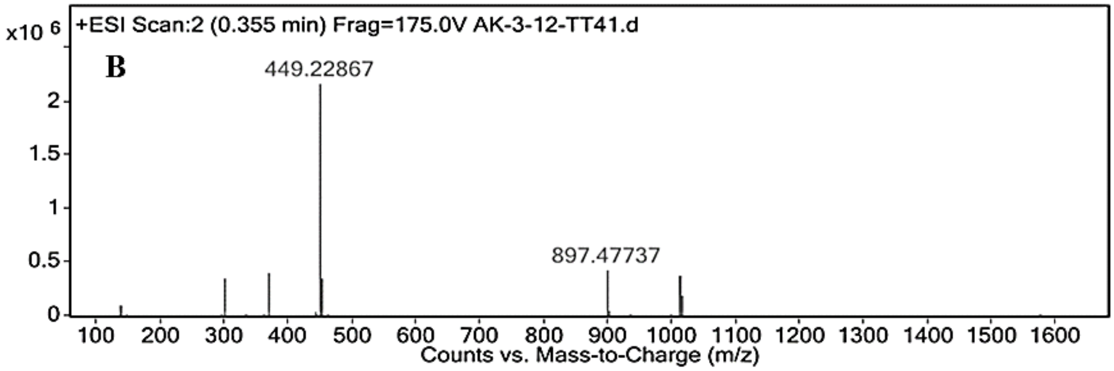

Supplementary Figure 9. *HPLC(A) and HRMS (B) spectra of KA311*

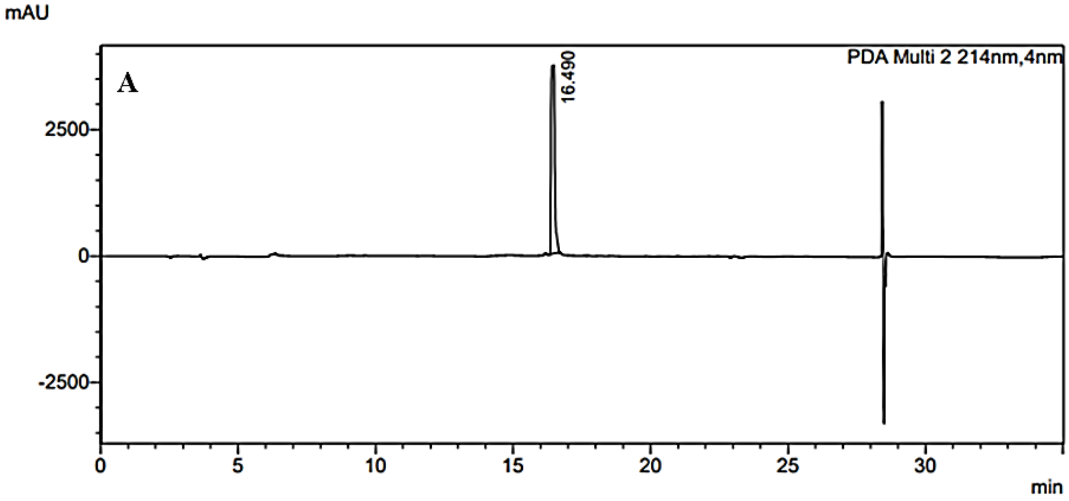

<Peak Table>

PDA Ch2 214nm

Ret. Time	Area	Area%
16.490	34583794	100.000
	34583794	100.000

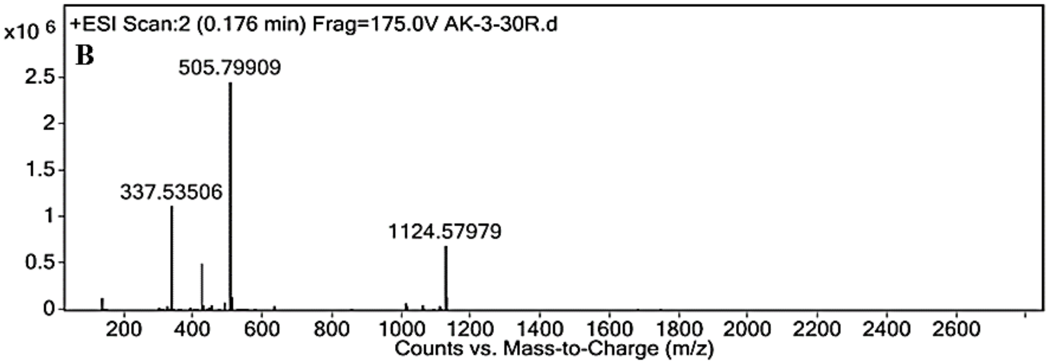

Supplementary Figure 10*. HPLC(A) and HRMS (B) spectra of KA314*

1.2 Supplementary tables

Supplementary table 1a. *Sequences for 1st generation synthesised Dynorphin analogs*

KA101	Tyr	Gly	Gly	4NO2-Phe	Leu	Arg	Arg
KA102	Tyr	Gly	Gly	4MeOTyr	Leu	Arg	Arg
KA103	Tyr	Gly	Gly	2-NH2-Phe	Leu	Arg	Arg
KA104	Tyr	Gly	Gly	Phe	Leu	Arg	His
KA105	Tyr	Gly	Gly	Phe	Leu	Arg	Asn
KA106	Tyr	Gly	Gly	Phe	Leu	Arg	Cap-COOH
KA107	MeTyr	Gly	Gly	Phe	Leu	Arg	Cap-COOH
KA108	MeTyr	Sar	Gly	Phe	Leu	MeLys	Cap-COOH
KA109	Tyr	cis-Acha	Phe	Leu	Arg	Ala	---
KA110	Tyr	gaba	Phe	Leu	Arg	Ala	---
KA111	MeTyr	Gly	Gly	Phe	Leu	Lys	Cap-COOH
KA112	Tyr	Sar	Gly	Phe	Leu	Lys	Cap-COOH
KA113	MeTyr	Sar	Gly	Phe	Leu	Lys	Cap-COOH
KA114	MeTyr	cis-Acha	Phe	Leu	Arg	Ala	---
KA115	2-NH2-Phe	Gly	Gly	Phe	Leu	Arg	Arg
KA116	PheGly	Gly	Gly	Phe	Leu	Arg	Arg
KA117	Tyr	Arg	Leu	Phe	gaba	Arg	---
KA118	4Py	Gly	Gly	Phe	Leu	Arg	Arg
KA119	Tyr	Sar	Gly	Phe	Leu	MeLys	Cap-COOH
KA120	Tyr	Gly	Gly	Cit	Leu	Arg	Arg
KA121	Tyr	Gly	Gly	Phe	Leu	Cit	Arg
KA122	Tyr	Gly	Gly	Phe	Leu	Asn	Arg
KA123	Tyr	cis-Acha	Phe	Leu	Arg	Arg	---
KA124	Tyr	Phe	Leu	Gly	Arg	Arg	---
KA125	Tyr	Gly	Gly	Dab	Leu	Arg	Arg
KA126	Tyr	Sar	Gly	Phe	Leu	MeArg	Cap-COOH
KA127	MeTyr	Sar	Gly	Phe	Leu	MeArg	Cap-COOH
KA128	Tyr	Dab	Gly	Phe	Leu	Arg	Arg
KA129	Tyr	Gly	D-Ala	Phe	Leu	Arg	Arg
KA130	Tyr	Gly	Gly	Trp	Leu	Arg	Arg
KA131	Tyr	gaba	Phe	Leu	Arg	Arg	---
KA132	Tyr	Sar	Phe	Leu	Arg	Arg	---
KA133	Tyr	Dab	Phe	Leu	Arg	Arg	---
KA134	Tyr	Gly	Gly	TAl	Leu	Arg	Arg
KA135	Tyr	Gly	Gly	4-COOH-Phe	Leu	Arg	Arg
KA136	Tyr	Sar	Gly	Phe	Leu	Arg	Cap-COOH
KA137	MeTyr	Sar	Gly	Phe	Leu	Arg	Cap-COOH
KA138	Tyr	Gly	Gly	His	Leu	Arg	Arg

KA139	Tyr	4Py	Leu	Gly	Arg	Arg	---
KA140	Tyr	TAl	Leu	Gly	Arg	Arg	---
KA141	TAl	Gly	Gly	Phe	Leu	Arg	Arg
KA142	H-B-Tyr	Gly	Gly	Phe	Leu	Arg	Arg
KA143	Tyr	Gly	Gly	Phe	Leu	Lys	Cap-COOH
KA144	MeTyr	cis-Acha	Phe	Leu	Arg	Arg	---
KA145	Tyr	gaba	Phe	Leu	Arg	Val	---
KA146	Tyr	Gly	Gly	Phe	Leu	Arg	H-Arg

Supplementary table 1b. *Sequences for 2nd generation synthesised Dynorphin analogs*

KA201	Tyr	Gly	Gly	p-NO2-Phe	Leu	Arg	D-Arg
KA202	Tyr	Sar	Gly	p-NO2-Phe	Leu	Arg	D-Arg
KA203	Tyr	Sar	Phe	Leu	Arg	D-Arg	
KA204	Tyr	Sar	p-NO2-Phe	Leu	Arg	D-Arg	
KA205	Tyr	Sar	p-NO2-Phe	Leu	D-Arg	Arg	---
KA206	Tyr	Sar	p-Cl-Phe	Leu	Arg	D-Arg	---
KA207	Tyr	Sar	p-F-Phe	Leu	Arg	D-Arg	---
KA208	Tyr	Gly	Sar	p-NO2-Phe	Leu	Arg	D-Arg
KA209	Tyr	Sar	Sar	p-NO2-Phe	Leu	Arg	D-Arg
KA210	Tyr	Sar	Sar	p-NO2-Phe	Leu	D-Arg	Arg
KA211	Tyr	Gly	Sar	p-NO2-Phe	Leu	D-Arg	Arg
KA212	Tyr	Sar	Gly	p-NO2-Phe	Leu	D-Arg	Arg
KA213	Tyr	Gly	Gly	p-NO2-Phe	Leu	D-Arg	Arg
KA214	Tyr	Sar	Gly	p-NO2-Phe	Leu	D-Arg	D-Arg
KA215	Phe	Sar	Sar	p-NO2-Phe	Leu	D-Arg	Arg
KA216	Phe	Gly	Sar	p-NO2-Phe	Leu	D-Arg	Arg
KA217	Phe	Sar	Sar	p-NO2-Phe	Leu	D-Lys	Arg
KA218	Tyr	Sar	Phe	Leu	D-Arg	Arg	---
KA219	Tyr	Sar	Phe	Leu	D-Arg	D-Arg	---
KA220	Phe	Sar	p-NO2-Phe	Leu	D-Arg	D-Arg	---
KA221	Phe	Sar	p-NO2-Phe	Leu	D-Lys	D-Arg	---
KA222	Tyr	Sar	p-NO2-Phe	Leu	D-Lys	D-Arg	---
KA223	Tyr	Sar	p-NO2-D-Phe	Leu	Arg	D-Arg	---
KA224	Tyr	Sar	p-Cl-D-Phe	Leu	Arg	D-Arg	---
KA225	Tyr	Sar	p-F-D-Phe	Leu	Arg	D-Arg	---

Supplementary table 1c. *Sequences for3rd generation synthesised Dynorphin analogs*

KA301	Tyr	Sar	p-Cl-Phe	Leu	NMA	D-Arg	---
KA302	Tyr	Sar	p-F-Phe	Leu	NMA	D-Arg	---
KA303	Tyr	Sar	p-NO2-Phe	Leu	NMA	D-Arg	---
KA304	Tyr	Sar	p-Cl-Phe	Leu	Arg	D-Arg	-C5H10-CONH2
KA305	Tyr	Sar	p-Cl-Phe	Leu	Arg	D-Arg	-C7H14-CONH2
KA306	Tyr	Sar	p-Cl-Phe	Leu	Lys	D-Arg	---
KA307	Tyr	Ala	p-Cl-Phe	Leu	Arg	D-Arg	---
KA308	Tyr	Sar	p-Cl-Phe	Thr	Arg	D-Arg	---
KA309	Tyr	Sar	p-Cl-Phe	Leu	Arg	NMA	---
KA310	Tyr	Sar	p-NO2-Phe	Leu	Arg	NMA	---
KA311	Tyr	Sar	p-NO2-Phe	Leu	NMA	NMA	---
KA312	Tyr	Sar	p-Cl-Phe	Leu	Arg	NMA	-C7H14-CONH2
KA313	Tyr	Sar	p-NO2-Phe	Leu	Arg	NMA	-C7H14-CONH2
KA314	Tyr	Sar	p-NO2-Phe	Leu	Arg	D-Arg	-C7H14-CONH2

Supplementary table 2. *Competitive binding of test ligands versus reference ligand Naltrindole in the HTRF assay. IC_{50} determined from data presented in Figure 5, K_i determined from IC_{50} and K_d and concentration of Naltrindole used for competitive binding (0.8nM and 8nM respectively)*

**Compound**	**Log IC_{50}**	**IC_{50} (nM)**	**Ki (nM)**
**Morphine**	-4.91	12,240.0	1,130.4
**U50488H**	-6.07	857.9	79.2
**Dyn 1-17**	-6.78	165.7	15.3
**Dyn 1-7**	-6.60	253.5	23.4
**KA204**	-7.52	30.2	2.8
**KA305**	-7.14	72.6	6.7
**KA311**	-5.71	1,953.0	180.4
